# Homeostasis in networks with multiple inputs

**DOI:** 10.1007/s00285-024-02117-5

**Published:** 2024-06-20

**Authors:** João Luiz de Oliveira Madeira, Fernando Antoneli

**Affiliations:** 1 Department of Mathematical Sciences, University of Bath, Bath, BA2 7AY UK; 2 Escola Paulista de Medicina, Universidade Federal de São Paulo, São Paulo, 04039-032 Brazil

**Keywords:** Homeostasis, Coupled systems, Combinatorial matrix theory, Input–output networks, Biochemical networks, Perfect adaptation, 92C42 (primary), 92C45, 37N25

## Abstract

Homeostasis, also known as adaptation, refers to the ability of a system to counteract persistent external disturbances and tightly control the output of a key observable. Existing studies on homeostasis in network dynamics have mainly focused on ‘perfect adaptation’ in deterministic single-input single-output networks where the disturbances are scalar and affect the network dynamics via a pre-specified input node. In this paper we provide a full classification of all possible network topologies capable of generating infinitesimal homeostasis in arbitrarily large and complex multiple inputs networks. Working in the framework of ‘infinitesimal homeostasis’ allows us to make no assumption about how the components are interconnected and the functional form of the associated differential equations, apart from being compatible with the network architecture. Remarkably, we show that there are just three distinct ‘mechanisms’ that generate infinitesimal homeostasis. Each of these three mechanisms generates a rich class of well-defined network topologies—called *homeostasis subnetworks*. More importantly, we show that these classes of homeostasis subnetworks provides a topological basis for the classification of ‘homeostasis types’: the full set of all possible multiple inputs networks can be uniquely decomposed into these special homeostasis subnetworks. We illustrate our results with some simple abstract examples and a biologically realistic model for the co-regulation of calcium ($\textrm{Ca}$) and phosphate ($\textrm{PO}_4$) in the rat. Furthermore, we identify a new phenomenon that occurs in the multiple input setting, that we call *homeostasis mode interaction*, in analogy with the well-known characteristic of multiparameter bifurcation theory.

## Introduction

A homeostatic process is characterized by the following property: approximately zero steady-state error to external disturbance, which means that an observable of interest is tightly controlled. Homeostasis is biologically important because it protects organisms against changes induced by the environment. A familiar example is thermoregulation, where the body temperature of an organism remains roughly constant despite variations in its environment (Morrison 1946). Another example is a biochemical reaction network, where the equilibrium concentration of some important molecule might not change much while the concentration of another reactant changes (Reed et al. 2017). Further examples include regulation of cell number and size (Lloyd 2013), sleep control (Wyatt et al. 1999), and expression level regulation of housekeeping genes (Antoneli et al. 2018).

Homeostasis can be mathematically defined as follows (see Sect. 2.1). Consider a dynamical system depending on an external parameter ${\mathcal {I}}$ which varies over an open interval $]{\mathcal {I}}_{1}, {\mathcal {I}}_{2}[$ of external stimuli. Suppose there is a family of equilibrium points $X({\mathcal {I}})$ and an observable $\phi$ such that the *input–output function* $z({\mathcal {I}})=\phi (X({\mathcal {I}}))$ is well-defined on $]{\mathcal {I}}_{1}, {\mathcal {I}}_{2}[$. In this situation, we say that the system exhibits *homeostasis* if, under variation of the external parameter ${\mathcal {I}}$, the input–output function $z({\mathcal {I}})$ remains ‘approximately constant’ over the interval of external stimuli.

There are two formulations of ‘approximately constant’ often considered by researchers. The first, more stringent, called *perfect homeostasis*, is widely studied in control engineering and synthetic biology under the name ‘perfect adaptation’ [cf. Mello and Tu (2003); Ma et al. (2009); Ang and McMillen (2013); Araujo and Liotta (2018); Khammash (2021); Frei and Khammash (2021)]. *Perfect homeostasis* is defined as the ability of a system to reset to its pre-stimulated output level, called the *set point*, after responding to arbitrary external stimuli. It is obvious that this condition is equivalent to the requirement that the input–output function is identically constant.

The second, more general, called *near-perfect homeostasis*, requires that the input–output function stays within a ‘narrow’ range under variation of external stimuli over a bounded interval. Hence, a typical ‘plot’ of the input–output function has a bounded region of homeostasis where it is approximately constant, called the *plateau*, flanked by regions of *escape from homeostasis*, where it varies monotonically. See plots of input–output functions fitting data sets sampled from real biological systems in Morrison (1946), Golubitsky and Stewart (2017b), Nijhout et al. (2018).

The notion of near-perfect homeostasis has appeared in the literature under the names *near-perfect adaptation* (Mello and Tu 2003; Ang and McMillen 2013; Ferrell 2016) and *imperfect adaptation* (Bhattacharya et al. 2022, 2021). A refinement of the notion of near-perfect homeostasis, called *infinitesimal homeostasis* has been proposed by Golubitsky and Stewart (Golubitsky and Stewart 2017b). Since then, aspects of this new concept have been explored in several publications (Reed et al. 2017; Golubitsky and Stewart 2018; Duncan et al. 2018; Duncan and Golubitsky 2019; Golubitsky and Wang 2020; Wang et al. 2021; Madeira and Antoneli 2022).

In this paper, we shall study near-perfect adaptation from the point of view of infinitesimal homeostasis theory. Other contributions on near-perfect homeostasis include (Bhattacharya et al. 2023; Blanchini et al. 2022; Gross et al. 2019) and references therein. These three groups propose distinct approaches to near-perfect homeostasis, under various assumptions on the functional form of the dynamics. Moreover, since all these approaches are distinct from the one presented here, it would be a too large detour for us to try to explain and compare all these ideas. Nevertheless, we believe that a review unifying all these ideas would an extremely valuable achievement that could bring closer the several groups working in this subject under different viewpoints.

We say a system with input–output function $z({\mathcal {I}})$ exhibits *infinitesimal homeostasis* if $\frac{dz}{d {\mathcal {I}}}({\mathcal {I}}_{0}) = 0$ for some input value ${\mathcal {I}}_{0}\in ]{\mathcal {I}}_{1},{\mathcal {I}}_{2}[$. The vanishing of the derivative of *z* at ${\mathcal {I}}_{0}$ implies that ${\mathcal {I}}_{0}$ is a *critical point* of *z*. Moreover, the second order derivative of *z* with respect to ${\mathcal {I}}_{0}$ can be used to give a quantitative estimate on the size of the interval $]{\mathcal {I}}_{1}, {\mathcal {I}}_{2}[$ where $z({\mathcal {I}})$ stays within $z({\mathcal {I}}_0)\pm \delta$, for a given $\delta >0$ (see Golubitsky and Stewart (2022) for details). As we shall see in a moment, there are some additional advantages in adopting this point of view, besides providing a plausible notion of near-perfect homeostasis.

Nijhout, Reed, and Best (Nijhout et al. 2004, 2015, 2018; Best et al. 2009; Nijhout and Reed 2014; Nijhout et al. 2017, 2019) among others, have shown that homeostasis is an important phenomenon in biochemical reaction networks. In a biochemical network, each node represents the concentration of a chemical substrate and each arrow denotes a chemical interaction between the molecules at the head and tail of the arrow. In an input–output network formulation, one node is designated as the input node $\iota$ and another is designated as the output node *o*. The modeling assumes that some external stimuli (represented by an *input parameter*, or simply an *input*, ${\mathcal {I}}$) affects the network dynamics only at the input node, and the end result of computation by the network dynamics is the value of the output node. In this setting, there is a canonical choice for the smooth observable $\phi$: the coordinate function of the output node.

Motivated by these examples, Wang et al. (2021) introduced the notion of ‘abstract input–output network’ and devised a scheme for the classification of ‘homeostasis types’ in such networks. The notion of homeostasis type of a network makes precise the idea that homeostasis may be caused by different ‘mechanisms’ in that network. The results of Wang et al. (2021) apply to the case of single-input single-output networks where the external stimuli can only affect one input node via a single scalar input.

Even though single-input single-output (SISO) networks are quite popular in many engineering domains (Ma et al. 2009; Ang and McMillen 2013; Araujo and Liotta 2018; Bhattacharya et al. 2022), the single input node and single input assumptions seem unrealistic in biology, as disturbances that arise are typically very complex and do not have a single well-defined entry point (Gupta and Khammash 2022). As far as the input is concerned, there are two possible ways to extend the work of Wang et al. (2021) in order to include more complex situations:

1. *Multiple input nodes.* A single input affects more than one of several input nodes.
2. *Multiple inputs.* Several inputs affect more than one of several input nodes.

Regarding (1), Madeira and Antoneli (2022) extended the classification of Wang et al. (2021) to the setting of multiple input nodes and used this extended theory to completely work out the homeostasis types of a representative model for bacterial chemotaxis (Clausznitzer et al. 2010; Tindall et al. 2008). As for (2), an interesting biologically relevant example is the regulation of extracellular dopamine (eDA) in response to variation in the activities of the enzyme tyrosine hydroxylase (TH) and the dopamine transporters (DAT) (Best et al. 2009; Golubitsky and Stewart 2018). Another biologically relevant example of the second situation is the mathematical model of Granjon et al. (2017) for the physiological co-regulation of calcium ($\textrm{Ca}$) and phosphate ($\textrm{PO}_4$) in the rat. We will discuss this model in details in Sect. 3.

Let us recall the results of Madeira and Antoneli (2022). The main discovery of Wang et al. (2021) is that, in a given abstract input–output network, there is a finite number of ‘distinct mechanisms’ that may cause homeostasis, i.e. may force the derivative of the input–output function to vanish (at a fixed value of the input). These ‘distinct mechanisms’, called ‘homeostasis types’, bijectively correspond to a specific subnetworks of the abstract input–output network. These subnetworks, called *homeostasis subnetworks*, can be characterized in purely topological terms. In the single-input single-output theory of Wang et al. (2021), the homeostasis subnetworks can be divided into two classes: *structural* and *appendage*. The structural subnetworks correspond to feedforward mechanisms and the appendage subnetworks correspond to feedback mechanisms. These are called, respectively, ‘opposer modules’ and ‘balancer modules’ in the control theoretic literature (Araujo and Liotta 2018). In Madeira and Antoneli (2022) the single-input single-output theory is generalized, for the first time, to account for multiple input nodes. The main result is that everything from the single-input case generalizes, except that there is a new class of homeostasis subneworks, called *input counterweight*: every abstract multiple input node network has exactly one input counterweight subnetwork, which can be topologically characterized, as well.

In this paper we further build on the theory of Wang et al. (2021); Madeira and Antoneli (2022) to completely solve the problem of classifying the homeostasis types for input–output networks with multiple input nodes and multiple inputs (Sect. 2.3). More precisely, given an input–output network with multiple inputs we show that one can consider each parameter at a time. Thus, effectively reducing the problem of classification of homeostasis types to the single input case (still with multiple input nodes), that has been completely solved in Madeira and Antoneli (2022). Afterwards, we show how to combine these partial classifications for the single input cases into an algorithm that provides the full classification on the multiple inputs setting (Sect. 2.8).

The first issue to consider in the multiple inputs case is to obtain an analogue of the ‘determinant formula’ for the gradient of the inputs-output function (cf. Golubitsky and Stewart 2017b; Golubitsky et al. 2020; Wang et al. 2021; Madeira and Antoneli 2022). Versions of this ‘determinant formula’ have been obtained by several authors: Ma et al. (2009), Golubitsky and Wang (2020) for three-node networks and Araujo and Liotta (2018), Aoki et al. (2019) for arbitrary networks under the name ‘RPA equation’. In Sect. 2.3 we introduce a definition of a multiple-input single-output network and prove a multivariate generalization of ‘determinant formula’ (Lemma 2.1) mentioned above. A similar result to our ‘determinant formula’ has been obtained in Tang and McMillen (2016).

The main result of this paper allows us to completely classify homeostasis subnetworks of multiple inputs ‘core’ network (Sects. 2.4, 4.1). In the multiple inputs setting two new features arise. The first is related to being able to consider one parameter at a time. More specifically, for each scalar input ${\mathcal {I}}_M$ we define a unique subnetwork, called ${\mathcal {I}}_M$-*specialized subnetwork*, that contains all the homeostasis subnetworks associated to the input ${\mathcal {I}}_M$ (Sect. 2.5). When we have all the homeostasis subneworks of the specialized subnetworks we can proceed by considering two cases: (i) a *pleiotropic subnetwork* if it appears in all ${\mathcal {I}}_M$-specialized subnetworks, (ii) a *coincidental subnetwork* otherwise. Then we show that a pleiotropic subnetwork can only be of structural or appendage classes (Sects. 2.6, 4.2), whereas a coincidental subnetwork can be of structural, appendage or input counterweight class (Sect. 2.7). Furthermore, all our results hold generically in the setting of *influence networks*, which includes all ODE models used in biology (see Sect. 2.2).

The second new feature of multiparameter setting is related to the occurrence of overlapping between coincidental subnetworks contained in distinct ${\mathcal {I}}_M$-specialized subnetworks. These non-trivial interactions between homeostasis subnetworks in multiple inputs networks leads to the appearence of *homeostasis mode interaction* or *higher codimension homeostasis*. The notion of mode interaction is familiar in bifurcation theory. In a steady-state bifurcation the eigenvectors of the linearized equation corresponding to simple eigenvalues are called *modes*. A mode whose eigenvalues lie on the imaginary axis is said to be *critical*. Generically, it is expected that an one-parameter system has only one critical mode. However, in systems with more than one parameter, one expects multiple critical modes. The steady-state bifurcations that may arise in nonlinear systems near a (multi-) parameter value at which there are multiple critical modes are thought of as resulting from a nonlinear interaction of several critical modes. This process is called *mode interaction* and the (multi-) parameter values at which there are multiple critical modes are called *higher codimension bifurcation points*. We assert that there is an analogue process in the context of infinitesimal homeostasis (Ex. 2.16). Duncan et al. (2023) investigates the appearance of codimension-two homeostasis mode interaction in the different setting of infinitesimal homeostasis with a single input parameter.

We end this introduction by briefly mentioning other aspects of the infinitesimal homeostasis approach that we will not pursue in this paper: singularity theory network-preserving changes of coordinates (Golubitsky and Stewart 2017a, b, 2018; Antoneli and Stewart 2022), biological robustness (Kitano 2004, 2007a, b; Khammash 2016; Thom 1969, 1975, 1977; Hunt et al. 1992; Ott and Yorke 2005), numerical discovery and continuation (Govaerts 2000; Ermentrout 2002; Donovan 2019), classification of infinitesimal homeostasis in small ‘core networks’ (Huang and Golubitsky 2022), infinitesimal homeostasis for limit cycles (Yu and Thomas 2022) and miscellaneous applications to biology (Antoneli et al. 2018; Madeira and Antoneli 2022; Golubitsky et al. 2020; Golubitsky and Wang 2020).

**Structure of the Paper.** In Sect. 2 we give the definitions and state the main results of the paper. We discuss some simple abstract examples to illustrate the definition and results. In Sect. 3 we apply our results to a ‘real biological system’, a mathematical model of calcium and phosphate metabolism proposed in Granjon et al. (2017). In Sect. 4 we give the proofs of all our results. Finally, in Sect. 5 we briefly discuss our results in the context of the theory of infinitesimal homeostasis and conclude the paper with an outlook for future research.

## Homeostasis in multiple inputs networks

In this section we state the main results of the paper and provide the necessary definitions.

### Dynamical theory of infinitesimal homeostasis

Golubitsky and Stewart proposed a mathematical method for the study of homeostasis based on dynamical systems theory (Golubitsky and Stewart 2017b, 2018) [see the review (Golubitsky et al. 2020)]. In this framework, one consider a system of differential equations

2.1 $$\begin{aligned} {\dot{X}} = F(X, {\mathcal {I}}) \end{aligned}$$

where $X = (x_{1}, \ldots , x_{n}) \in {\mathbb {R}}^{n}$ is the vector of state variables and ${\mathcal {I}}=({\mathcal {I}}_1,\ldots ,{\mathcal {I}}_N)\in {\mathbb {R}}^{N}$ is the vector of input parameters.

Suppose that $(X^*, {\mathcal {I}}^*)$ is a linearly stable equilibrium of (2.1). By the implicit function theorem, there is a function ${\tilde{X}}({\mathcal {I}})$ defined in a neighborhood of ${\mathcal {I}}^*$ such that ${\tilde{X}}({\mathcal {I}}^*) = X^*$ and $F({\tilde{X}}({\mathcal {I}}), {\mathcal {I}}) \equiv 0$. See Jahedi et al. (2022) for results on the generic existence and robustness of ${\tilde{X}}({\mathcal {I}})$.

A smooth function $\phi :{\mathbb {R}}^{n}\rightarrow {\mathbb {R}}$ is called an *observable*. Define the *input–output function* $z:{\mathbb {R}}^{N}\rightarrow {\mathbb {R}}$ associated to $\phi$ and ${\tilde{X}}$ as $z({\mathcal {I}})=\phi ({\tilde{X}}({\mathcal {I}}))$. The input–output function allows one to formulate several definitions that capture the notion of homeostasis (see Ma et al. 2009; Ang and McMillen 2013; Tang and McMillen 2016; Golubitsky and Stewart 2017b, 2018).

#### Definition 2.1

Let $z({\mathcal {I}})$ be the input–output function associated to a system of differential Eqs. (2.1). We say that $z({\mathcal {I}})$ exhibits

- *Perfect Homeostasis* on an open set $\Omega \subseteq {\text {dom}}(z)$ if
  2.2 $$\begin{aligned} \nabla z({\mathcal {I}}) = 0 \qquad \text {for all} \; {\mathcal {I}} \in \Omega \end{aligned}$$
  That is, *z* is constant on $\Omega$.
- *Near-perfect Homeostasis* relative to a *set point* ${\mathcal {I}}_{\textrm{s}} \in \Omega \subseteq {\text {dom}}(z)$ if, for fixed $\delta$,
  2.3 $$\begin{aligned} | z({\mathcal {I}}) - z({\mathcal {I}}_{\textrm{s}}) | \leqslant \delta \qquad \text {for all} \; {\mathcal {I}} \in \Omega \end{aligned}$$
  That is, *z* stays within the range $z({\mathcal {I}}_{\textrm{s}})\pm \delta$ over $\Omega$.
- *Infinitesimal Homeostasis* at the point ${\mathcal {I}}_{\textrm{c}} \in {\text {dom}}(z)$ if
  2.4 $$\begin{aligned} \nabla z({\mathcal {I}}_{\textrm{c}}) = 0 \end{aligned}$$
  That is, ${\mathcal {I}}_{\textrm{c}}$ is a *critical point* of *z*. $\Diamond$

It is clear that perfect homeostasis implies near-perfect homeostasis, but the converse does not hold. Inspired by Nijhout, Reed, Nijhout et al. (2004); Best et al. (2009); Golubitsky and Stewart (2017b, 2018) introduced the notion of infinitesimal homeostasis that is intermediate between perfect and near-perfect homeostasis. It is obvious that perfect homeostasis implies infinitesimal homeostasis. On the other hand, it follows from Taylor’s theorem that infinitesimal homeostasis implies near-perfect homeostasis in a neighborhood of ${\mathcal {I}}_{\textrm{c}}$, see Golubitsky and Stewart (2022). It is easy to see that the converse to both implications is not generally valid (see Reed et al. 2017).

The notion of infinitesimal homeostasis allows one to apply the tools from singularity theory. For instance, by considering higher degeneracy conditions, in addition to (2.4), one is lead to distinct forms of infinitesimal homeostasis that can be classified by elementary catastrophe theory (see Golubitsky and Stewart 2017b, 2018 for details). Finally, when combined with coupled systems theory (Golubitsky and Stewart 2006) the formalism of Golubitsky and Stewart (2017b), Golubitsky and Stewart (2018), Golubitsky et al. (2020) becomes very effective in the analysis of model equations.

### Networks and dynamical systems

Before defining the appropriate class of dynamical systems (in Sect. 2.3) for our results we will briefly discuss the relation between networks and dynamics.

A large portion of the literature on network dynamical systems modeling seems to suggest that there is a unique way to associate a system of differential equations to a given directed graph ${\mathcal {G}}$. With some rare exceptions, e.g. Bick et al. (2023), a precise definition of what is a “network dynamical system” is completely overlooked and the *pairwise interaction interpretation* (see below) is assumed without further justification. In fact, there are at least, *two* possible ways to attach a (class of) dynamical system(s) to a directed graph ${\mathcal {G}}$. In order to discuss the distinction between these two possibilities we need precise definitions. Let ${\mathcal {G}}$ be a directed graph with *k* nodes.

1. *Pairwise interaction interpretation.* In this interpretation the dynamics is encoded by a weighted adjacency matrix *A* compatible with the directed graph ${\mathcal {G}}$. That is, $A_{ji} \ne 0$ if and only if there is a link from node *j* to node *i*. For simplicity, suppose that each node of ${\mathcal {G}}$ represent an identical dynamical system of the form ${\dot{x}}_i = F(x_i)$, where $x_i\in {\mathbb {R}}^n$ is the *state vector* of node *i* and $F:{\mathbb {R}}^n\rightarrow {\mathbb {R}}^n$ is a smooth function that describes the *internal dynamics* of node *i*. The *interaction* between nodes is given by a smooth *pairwise coupling function* $G:{\mathbb {R}}^n\times {\mathbb {R}}^n\rightarrow {\mathbb {R}}^n$, also called *point-to-point coupling* (Golubitsky and Stewart 2002). The tuple (*A*, *F*, *G*) defines a *network dynamical system* through the set of differential equations
  2.5 $$\begin{aligned} {\dot{x}}_i = F(x_i) + \sum _{j=1}^k A_{ji}G(x_i,x_j) \qquad \text {for}\quad i = 1,\dots ,k \end{aligned}$$
  The dynamics is determined by the evolution of the joint state of all nodes $(x_1, \ldots , x_k)$ through (2.5). It is important to note that network dynamical systems described via (2.5) have only *additive interactions*. Specifically, the interactions are in general nonlinear in the state variables $x_i$, but *linear* in the coupling weights $A_{ji}$Aguiar and Dias (2018). By letting *F* and *G* vary over all smooth functions on the state space ${\mathbb {R}}^n$, for a given family of weighted adjacency matrix ${\mathcal {A}}$ defining the same directed graph ${\mathcal {G}}$, one obtains a space of vector fields ${\mathfrak {X}}^\infty _{\mathcal {A}}({\mathbb {R}}^n)$. While setup (2.5) is arguably one of the most commonly used formulations of network-based modeling, it imposes a severe restriction that might not hold in real-world systems (Bick et al. 2023).
2. *Influence network interpretation.* In this interpretation the dynamics is given by a smooth vector-valued function $H = (H_1, \ldots , H_k)$ that is compatible with the directed graph ${\mathcal {G}}$. That is, the component function $H_i$ explicitly depends on the state vector $x_j$ if and only if there is a link from node *j* to node *i*. Now the weights are implicitly incorporated into the function *H*. The dynamics is defined by the system of ODEs
  2.6 $$\begin{aligned} {\dot{x}}_i = F(x_i) + H_i(x_1,\ldots ,x_k) \qquad \text {for}\quad i = 1,\dots ,k \end{aligned}$$
  here $H_i:({\mathbb {R}}^{n})^k\rightarrow {\mathbb {R}}^n$ determines the *influence* of the joint state $(x_1,\ldots ,x_k)$ on the *i*-th node state $x_i$. The function $H_i$ may depend not only on two node states, but may involve multiple nodes concurrently. Network dynamical systems of the form (2.6) have been considered in the *groupoid formalism* (Golubitsky and Stewart 2006, 2022). Now, despite node dependencies being captured by a graph, this does not exclude the possibility of nonlinear interactions involving three or more nodes, called *higher-order interactions*. By letting *F* and *H* vary over all smooth functions on the state space ${\mathbb {R}}^n$ one obtains a space of vector fields ${\mathfrak {X}}^\infty _{{\mathcal {G}}}({\mathbb {R}}^n)$, this is the space of all dynamical systems (2.6) that are compatible with the network structure ${\mathcal {G}}$. The main goal of the groupoid formalism is to study the space ${\mathfrak {X}}^\infty _{{\mathcal {G}}}({\mathbb {R}}^n)$, rather than considering (2.6) for a specific *F* and *H*. This yields insights on how the ‘generic’ dynamical behavior of such a system depends on the imposed network structure encoded by ${\mathcal {G}}$.

It is easy to see that the space of vector fields ${\mathfrak {X}}^\infty _{{\mathcal {G}}}({\mathbb {R}}^n)$ contains the space of vector fields ${\mathfrak {X}}^\infty _{\mathcal {A}}({\mathbb {R}}^n)$, for all families of weighted adjacency matrices ${\mathcal {A}}$ defining the directed graph ${\mathcal {G}}$. In fact, ${\mathfrak {X}}^\infty _{{\mathcal {G}}}({\mathbb {R}}^n)$ is the *largest* space of vector fields that can be attached to a directed graph ${\mathcal {G}}$. Likewise, ${\mathfrak {X}}^\infty _{\mathcal {A}}({\mathbb {R}}^n)$, for a fixed ${\mathcal {A}}$, is the *smallest* space of vector fields that can be attached to a family of weighted adjacency matrix ${\mathcal {A}}$. Remarkably, it seems that in order to generalize (2.5) to include higher order interactions *only up to a fixed level*, directed graphs are not enough to capture all the interaction relations. It is necessary to consider ‘higher dimensional’ generalizations of graphs, such as *hypergraphs* or *simplicial complexes* (Bick et al. 2023).

In this paper we adopt the influence network interpretation. As mentioned before, this approach emphasizes the ‘generic’ properties and how they are affected by the network structure encoded by ${\mathcal {G}}$. This leads to the notion of *model-independent approach*, which is thoroughly explored in the book Golubitsky and Stewart (2022). The *model-independent approach* contrasts with the more common *model-dependent* one, where specific model equations are solved, usually numerically. Both approaches have advantages and disadvantages and should not be seen as competing against each other, on the contrary, they complement each other. Specific models are useful in connection with experimental tests, when the equations are obtained from precise laws. In a model-independent approach the exact equations may not be known, nevertheless one still can predict, from known properties of the equations, what types of behaviors are expected and which ones are forbidden.

### Multiple-input single-output networks

A *multiple-input single-output (MISO)* network, or simply a *multiple inputs network*, is a network ${\mathcal {G}}$ with *n* distinguished *input nodes* $\iota =\{\iota _{1}, \iota _{2}, \ldots , \iota _{n}\}$, all of them associated to at least one input parameter ${\mathcal {I}}_{M}$, $M = 1, \ldots , N$, one distinguished *output node* *o*, and *r* *regulatory nodes* $\rho =\{\rho _1,\ldots ,\rho _r\}$. The associated system of differential equations have the form

2.7 $$\begin{aligned} \begin{aligned} {\dot{x}}_{\iota }&= f_{\iota }(x_{\iota }, x_{\rho }, x_{o}, {\mathcal {I}}) \\ {\dot{x}}_{\rho }&= f_{\rho }(x_{\iota }, x_{\rho }, x_{o})\\ {\dot{x}}_{o}&= f_{o}(x_{\iota }, x_{\rho }, x_{o}) \end{aligned} \end{aligned}$$

where ${\mathcal {I}} = ({\mathcal {I}}_{1}, \cdots , {\mathcal {I}}_{N}) \in {\mathbb {R}}^{N}$ is the vector of *input parameters*, or simple the vector of *inputs*, and $X=(x_{\iota },x_{\rho },x_o)\in {\mathbb {R}}^n\times {\mathbb {R}}^r \times {\mathbb {R}}$ is the vector of state variables associated to the network nodes.

We write a vector field associated with the system (2.7) as

$$\begin{aligned} F(X,{\mathcal {I}})=(f_{\iota }(X,{\mathcal {I}}),f_\rho (X),f_o(X)) \end{aligned}$$

and call it an *admissible vector field* for the network ${\mathcal {G}}$.

Let $f_{j,x_i}$ denote the partial derivative of the $j^{th}$ node function $f_j$ with respect to the $i^{th}$ node variable $x_i$. We make the following assumptions about the vector field *F* throughout:

- The vector field *F* is smooth and has a linearly stable equilibrium at $(X^*,{\mathcal {I}}^*)$. Therefore, by the implicit function theorem, there is a function ${\tilde{X}}({\mathcal {I}})$ defined in a neighborhood of ${\mathcal {I}}^*$ such that ${\tilde{X}}({\mathcal {I}}^*) = X^*$ and $F({\tilde{X}}({\mathcal {I}}), {\mathcal {I}}) \equiv 0$.
- The partial derivative $f_{j,x_i}$ can be non-zero only if the network ${\mathcal {G}}$ has an arrow $i\rightarrow j$, otherwise $f_{j,x_i} \equiv 0$.
- Only the input node coordinate functions $f_{\iota _k}$ depend on at least one of the components of the vector of input parameters ${\mathcal {I}}$ and the partial derivative of $f_{\iota _k,{\mathcal {I}}_{M}}$ generically satisfies
  2.8 $$\begin{aligned} \frac{\partial f_{\iota _{k}}}{\partial {\mathcal {I}}_{M}} = f_{\iota _k,{\mathcal {I}}_{M}} \ne 0. \end{aligned}$$
  for some $$M = 1, \ldots , N$$.

#### Remark 2.2

In this paper we explicitly exclude the possibility that the output node is one of the input nodes. This assumption is included purely for the sake of convenience. In fact, all the results should be valid in this case, but then all the theorems and proofs should be properly adapted to take this particular case into account. This possibility, in the case of single input networks will be considered with great detail in another publication (Antoneli et al. 2023). $\Diamond$

The network structure provides a distinguished class of observables for an admissible system, namely, the state variables. In the particular case of an input–output network the observable of interest is given by the output state variable $x_o$.

#### Definition 2.3

Let ${\mathcal {G}}$ be a multiple inputs network and *F* be a family of admissible vector fields with an equilibrium point ${\tilde{X}}({\mathcal {I}})=\big (x_{\iota }({\mathcal {I}}),x_{\rho }({\mathcal {I}}),x_o({\mathcal {I}})\big )$. The mapping ${\mathcal {I}} \mapsto x_o({\mathcal {I}})$ is called the *input–output function* of the network ${\mathcal {G}}$, relative to the family of equilibria $\big ({\tilde{X}}({\mathcal {I}}),{\mathcal {I}}\big )$. $\Diamond$

Infinitesimal homeostasis in a multiple inputs network is given by the critical points of $x_o({\mathcal {I}})$, namely, the zeros of the gradient vector

2.9 $$\begin{aligned} \nabla x_{o} = \left( \frac{\partial x_{o}}{\partial {\mathcal {I}}_{1}}, \frac{\partial x_{o}}{\partial {\mathcal {I}}_{2}}, \cdots , \frac{\partial x_{o}}{\partial {\mathcal {I}}_{N}} \right) \end{aligned}$$

By a straightforward application of Cramer’s rule Wang et al. (2021) obtained a determinant formula for the derivative of the input–output function in the single-input single-output case. Madeira and Antoneli (2022) generalized the determinant formula of Wang et al. (2021) to the case of multiple input nodes networks. In the following we further generalize the determinant formula of Madeira and Antoneli (2022) to the case of multiple inputs networks.

Let *J* be the $(n+r+1)\times (n+r+1)$ Jacobian matrix of an admissible vector field $F=(f_{\iota },f_{\sigma },f_{o})$, that is,

2.10 $$\begin{aligned} J = \begin{pmatrix} f_{\iota , x_\iota } &{} \quad f_{\iota , x_\rho } &{} \quad f_{\iota , x_o} \\ f_{\rho , x_\iota } &{} \quad f_{\rho , x_\rho } &{} \quad f_{\rho , x_o} \\ f_{o, x_\iota } &{} \quad f_{o, x_\rho } &{} \quad f_{o, x_o} \end{pmatrix} \end{aligned}$$

For each $1 \le M \le N$, consider the $(n+r+1)\times (n+r+1)$ matrix $\langle H_M \rangle$ obtained from *J* by replacing the last column by $(-f_{\iota ,{\mathcal {I}}_M},0,0)^t$, is called ${\mathcal {I}}_M$-*generalized homeostasis matrix*:

2.11 $$\begin{aligned} \langle H_M \rangle = \begin{pmatrix} f_{\iota , x_\iota } &{} \quad f_{\iota , x_\rho } &{} \quad -f_{\iota , {\mathcal {I}}_M} \\ f_{\rho , x_\iota }&{} \quad f_{\rho , x_\rho } &{} \quad 0 \\ f_{o, x_\iota } &{} \quad f_{o, x_\rho } &{} \quad 0 \end{pmatrix} \end{aligned}$$

Here all partial derivatives $f_{\ell ,x_j}$ are evaluated at $\big ({\tilde{X}}({\mathcal {I}}),{\mathcal {I}}\big )$.

#### Lemma 2.1

Let $x_o({\mathcal {I}})$ be input–output function of a multiple inputs network. The partial derivative of $x_o({\mathcal {I}})$ with respect to the *M*-th parameter ${\mathcal {I}}_M$ satisfies

2.12 $$\begin{aligned} \frac{\partial x_o\;}{\partial {\mathcal {I}}_M} = \frac{\det \langle H_M \rangle }{\det (J)} \end{aligned}$$

where $\det (J)$ and $\det \langle H_M \rangle$ are evaluated at the equilibrium point ${\tilde{X}}({\mathcal {I}})$. Hence,

2.13 $$\begin{aligned} \nabla x_{o} = \frac{1}{\det (J)}\left( \det \langle H_{1} \rangle , \det \langle H_{2} \rangle , \ldots , \det \langle H_{N} \rangle \right) \end{aligned}$$

Moreover, ${\mathcal {I}}^0$ is a point of infinitesimal homeostasis if and only if

2.14 $$\begin{aligned} \det \langle H_M \rangle = 0 \qquad \text {for all} \quad 1 \le M \le N \end{aligned}$$

as a function of ${\mathcal {I}}$ evaluated at ${\mathcal {I}}^0$.

#### Proof

Implicit differentiation of the equation $F({\tilde{X}}({\mathcal {I}}),{\mathcal {I}})=0$ with respect to ${\mathcal {I}}$ yields the linear system

2.15 $$\begin{aligned} J \begin{pmatrix} x_i' \\ x_\rho ' \\ x_o'\end{pmatrix} = -\begin{pmatrix}f_{\iota , {\mathcal {I}}_M} \\ 0 \\ 0 \end{pmatrix} \end{aligned}$$

Since ${\tilde{X}}({\mathcal {I}})$ is assumed to be a linearly stable equilibrium, it follows that $\det (J)\ne 0$. On applying Cramer’s rule to (2.15) we can solve for $\frac{\partial x_o\;}{\partial {\mathcal {I}}_M}({\mathcal {I}})$ obtaining (2.12). Applying (2.12) to (2.9), we obtain Eq. (2.13). $\square$

#### Remark 2.4

An explicit expression for $\det \langle H_M \rangle$ can be obtained by expanding it with respect to the last column and the $\iota _m$-th row:

2.16 $$\begin{aligned} \det \langle H_M \rangle = \sum _{m=1}^n \pm f_{\iota _m,{\mathcal {I}}_M} \det (H_{\iota _m}) \end{aligned}$$

where $H_{\iota _m}$ is obtained from *H* by removing the last column and the $\iota _m$-th row. When there is a single input, i.e. $N=1$, the gradient $\nabla x_{o}$ reduces to ordinary derivative $x_o'$ and (2.13) gives the formula for $x_o'$ obtained in Madeira and Antoneli (2022). When there is a single input and a single input node, $N=n=1$, there is only one matrix $H_{\iota _m}=H$, called the *homeostasis matrix* and (2.13) gives the corresponding formula for $x_o'$ obtained in Wang et al. (2021). $\Diamond$

### Core networks

Wang et al. (2021) introduced a fundamental construction for the study of homeostasis in input–output networks, called ‘core subnetwork’. Madeira and Antoneli (2022) extended this construction to the case of input–output networks with multiple input nodes and single input parameter. Here we extend the arguments of Wang et al. (2021), Madeira and Antoneli (2022) to the case of multiple inputs networks.

Let $(X^*, {\mathcal {I}}^*)$ be a linearly stable equilibrium of the ${\mathcal {G}}$-admissible ODE system (2.1). Then $(X^*, {\mathcal {I}}^*)$ it satisfies the system of equations

2.17 $$\begin{aligned} \begin{aligned}&f_{\iota _{1}}(x_{\iota _{1}}, \ldots , x_{\iota _{n}}, x_{\rho }, x_{o}, {\mathcal {I}}_{1},\ldots , {\mathcal {I}}_{N}) = 0 \\&\qquad \qquad \qquad \vdots \\&f_{\iota _{n}}(x_{\iota _{1}}, \ldots , x_{\iota _{n}}, x_{\rho }, x_{o}, {\mathcal {I}}_{1},\ldots , {\mathcal {I}}_{N}) = 0 \\&f_{\rho }(x_{\iota _{1}}, \ldots , x_{\iota _{n}}, x_{\rho }, x_{o}) = 0 \\&f_{o}(x_{\iota _{1}}, \ldots , x_{\iota _{n}}, x_{\rho }, x_{o}) = 0 \end{aligned} \end{aligned}$$

Partition the nodes of the network ${\mathcal {G}}$ as follows: (i) input nodes (whose dynamics explicitly depends on at least one input), (ii) the output node and (iii) the regulatory nodes, that can be classified into three types depending if they are upstream from the output node or/and downstream from at least one input node. More precisely, the set of regulatory nodes may be partitioned as:

- Those nodes $\sigma$ that are both upstream from *o* and downstream from at least one input node $\iota _{m}$,
- Those nodes *d* that are not downstream from any input node $\iota _{m}$,
- Those nodes *u* which are downstream from at least one input node $\iota _{m}$, but not upstream from *o*.

**Fig. 1:**
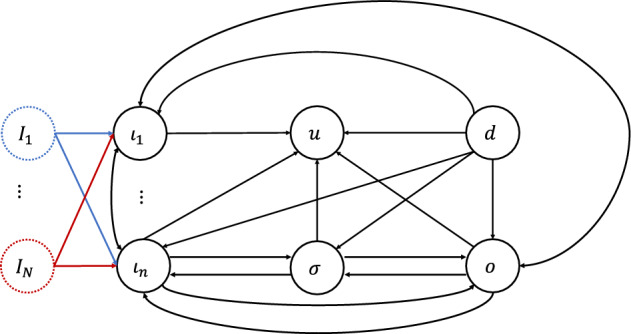
The possible connections in ${\mathcal {G}}$. Here, inputs are highlighted by dotted circles of distinct colors. The arrows from each input to distinct input nodes are of the same color (the same color of the corresponding dotted circle)

Figure 1 shows the types of connections which can be found in ${\mathcal {G}}$.

#### Definition 2.5

Let ${\mathcal {G}}$ be a multiple inputs network. The *core subnetwork* ${\mathcal {G}}_{c}$ of ${\mathcal {G}}$ is the subnetwork whose nodes are: (i) the input nodes $\iota _{1}, \ldots , \iota _{n}$, (ii) the regulatory nodes $\sigma$ that are upstream from the output node and downstream of at least one input node, and (iii) the output node *o*. The arrows of ${\mathcal {G}}_{c}$ are the arrows of ${\mathcal {G}}$ connecting the nodes of ${\mathcal {G}}_{c}$. $\Diamond$

#### Theorem 2.2

Let ${\mathcal {G}}$ be a multiple inputs network and ${\mathcal {G}}_c$ the corresponding core subnetwork. Then the input–output function $x^{c}_{o}$ of ${\mathcal {G}}_c$ exhibits infinitesimal homeostasis at ${\mathcal {I}}^*$ if and only if the input–output function $x_{o}$ of ${\mathcal {G}}$ exhibits infinitesimal homeostasis at ${\mathcal {I}}^*$.

#### Proof

Follows from Theorem 4.2. $\square$

Theorem 2.2 allows one to assume, without loss of generality, that ${\mathcal {G}}$ is a *core network*, that is ${\mathcal {G}}={\mathcal {G}}_c$, as far as infinitesimal homeostasis is concerned.

### Structure of infinitesimal homoestasis

In this subsection, unless explicitly stated, we assume that ${\mathcal {G}}$ is a core multiple inputs network with input nodes $\iota _{1}, \ldots , \iota _{n}$ and inputs ${\mathcal {I}}_{1}, \ldots , {\mathcal {I}}_{N}$.

By Lemma 2.1 a network ${\mathcal {G}}$ exhibits infinitesimal homeostasis at a point ${\mathcal {I}}^0$ whenever the vector-valued function (when evaluated at $({\tilde{X}}({\mathcal {I}}), {\mathcal {I}})$) vanishes at ${\mathcal {I}}^0$:

2.18 $$\begin{aligned} \vec {h}= \big (\det \langle H_{1} \rangle , \det \langle H_{2} \rangle , \ldots , \det \langle H_{N} \rangle \big ). \end{aligned}$$

here $\det \langle H_{M} \rangle$ are the determinants of the ${\mathcal {I}}_M$-generalized homeostasis matrices.

In order to analyze and simplify these determinants let us introduce some terminology. A *multivariate vector-valued polynomial*, or, simply a *polynomial mapping* is a mapping $P:{\mathbb {R}}^{k} \rightarrow {\mathbb {R}}^k$ with polynomial components. That is, if *P* is a polynomial mapping, there exist multivariate polynomials $P_{1}, P_{2}, \ldots , P_{k}:{\mathbb {R}}^{k}\rightarrow {\mathbb {R}}$ such that we can write *P* as

$$\begin{aligned} P(x_1,\ldots ,x_k)=\big ( P_1(x_1,\ldots ,x_k),\ldots , P_k(x_1,\ldots ,x_k) \big ). \end{aligned}$$

We say that *P* is irreducible if and only if each component $P_j$ is irreducible. Suppose there is a multivariate polynomial function $p:{\mathbb {R}}^{k} \rightarrow {\mathbb {R}}$ that is common factor to all components $P_j$. Then we can factor *p* from the polynomial vector *P* as

$$\begin{aligned} P(x_1,\ldots ,x_k)=p(x_1,\ldots ,x_k) \, \big ( {\tilde{P}}_1(x_1,\ldots ,x_k),\ldots , {\tilde{P}}_k(x_1,\ldots ,x_k) \big ). \end{aligned}$$

We say that *p* is a *scalar factor* of *P*.

Recall that the nonzero entries of the ${\mathcal {I}}_M$-generalized homeostasis matrices $\langle H_M \rangle$ are the partial derivatives $f_{j,x_i}$ and $f_{j,I_M}$. In particular, $\det \langle H_M \rangle$ is a homogeneous polynomial function of degree $(n+r+1)$ in the partial derivatives $f_{j,x_i}$ and $f_{j,I_M}$. Hence, the vector-valued function $\vec {h}$ is a (formal) polynomial mapping on the ‘variables’ $f_{j,x_i}$ and $f_{j,I_M}$. The scalar-valued function $\vec {h}({\mathcal {I}})$ (depending on the vector of input parameters ${\mathcal {I}}$) is obtained by evaluating the partial derivatives $f_{j,x_i}$ and $f_{j,I_M}$ at ${\tilde{X}}({\mathcal {I}})$.

**Fig. 2:**
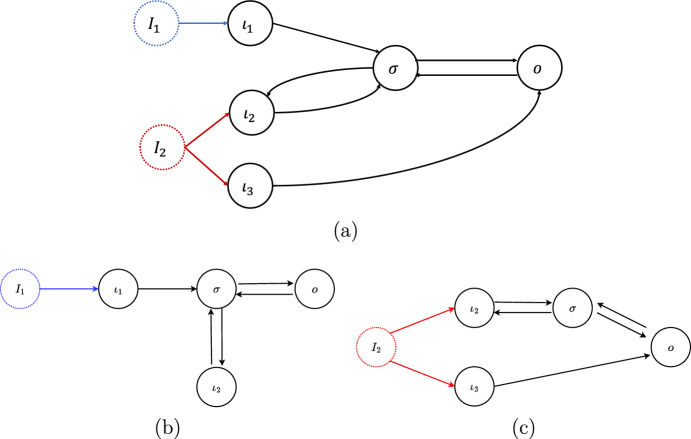
A 2-parameter 5-four node network (**a**) and its ${\mathcal {I}}_{M}$-specialized subnetworks (**b**) and (**c**). Here, inputs are highlighted by dotted circles of distinct colors. The arrows from each input to distinct input nodes are of the same color (the same color of the corresponding dotted circle). The 2-parameter network (**a**), has 5 nodes: three input nodes $\iota _1$, $\iota _2$, $\iota _3$, one output node *o* and one regulatory node $\sigma$. The two ${\mathcal {I}}_{M}$-*specialized subnetworks* of (**a**) are always single-input multiple input node networks (see Definition 2.6). The ${\mathcal {I}}_1$-specialized subnetwork (**b**) has 1 input node ($\iota _1$). Notice that $\iota _2$ becomes a regulatory node for this network. The ${\mathcal {I}}_2$-specialized subnetwork (**c**) has 2 input nodes ($\iota _2$, $\iota _3$)

Let us motivate the next definition with a simple observation. In the multiparameter setting, even a core network may have nodes that are not affected by all inputs. For example, consider the 5-node multiple inputs network ${\mathcal {G}}$ shown in Fig. 2. In this figure inputs are highlighted by dotted circles of distinct colors. The arrows from each input to distinct input nodes are of the same color (the same color of the corresponding dotted circle). The network shown in (a) has three input nodes, $\iota _1$, $\iota _2$ and $\iota _3$, and two inputs, ${\mathcal {I}}_{1}$ (blue) and ${\mathcal {I}}_{2}$ (red). Although, ${\mathcal {G}}$ is a core network, the input node $\iota _{1}$ is not affected by the parameter ${\mathcal {I}}_{2}$, and the node $\iota _{3}$ is not affected by parameter ${\mathcal {I}}_{1}$. To overcome this difficulty, we define the ‘specialized networks’ relative to a single input parameter.

#### Definition 2.6

Let ${\mathcal {G}}$ be a core multiple inputs network with inputs ${\mathcal {I}}_{1}, \ldots , {\mathcal {I}}_{N}$. The ${\mathcal {I}}_{M}$-*specialized subnetwork* ${\mathcal {G}}_{{\mathcal {I}}_{M}}$ is defined as the (single input) input–output subnetwork of ${\mathcal {G}}$ consisting of all the input nodes that receive the input ${\mathcal {I}}_{M}$, all the regulatory nodes that are downstream from those input nodes and the output node. The arrows of ${\mathcal {G}}_{{\mathcal {I}}_{M}}$ are the arrows of ${\mathcal {G}}$ between the nodes of ${\mathcal {G}}_{{\mathcal {I}}_{M}}$. The subnetwork ${\mathcal {D}}_{{\mathcal {I}}_{M}}={\mathcal {G}} {\setminus } {\mathcal {G}}_{{\mathcal {I}}_M}$ generated by the nodes in ${\mathcal {G}}$ that do not belong to ${\mathcal {G}}_{{\mathcal {I}}_{M}}$ is called the ${\mathcal {I}}_{M}$-*vestigial subnetwork*. $\Diamond$

The specialized subnetwork ${\mathcal {G}}_{{\mathcal {I}}_{M}}$ can be considered as a multiple input node network with single input ${\mathcal {I}}_M$, as studied in Madeira and Antoneli (2022), by ‘forgetting’ the effect of the other parameters and reducing to the core network using the core reduction theorem of (Madeira and Antoneli 2022, Thm. 3.2). The input nodes of ${\mathcal {G}}_{{\mathcal {I}}_{M}}$ are exactly the input nodes of ${\mathcal {G}}$ that are affected by the parameter ${\mathcal {I}}_{M}$.

#### Definition 2.7

Let ${\mathcal {G}}$ be a core multiple inputs network with inputs ${\mathcal {I}}_{1}, \ldots , {\mathcal {I}}_{N}$. The ${\mathcal {I}}_M$-*homeostasis matrix* $\langle H_{{\mathcal {I}}_M} \rangle$ associated to ${\mathcal {G}}_{{\mathcal {I}}_{M}}$, is the generalized homeostasis matrix of the multiple input–output network ${\mathcal {G}}_{{\mathcal {I}}_{M}}$ (see Madeira and Antoneli (2022, Sec 2.3)). Similarly, the corresponding ${\mathcal {I}}_M$-vestigial subnetwork ${\mathcal {D}}_{{\mathcal {I}}_{M}}$ has an associated *jacobian matrix* $J_{{\mathcal {D}}_{{\mathcal {I}}_{M}}}$ (see Madeira and Antoneli (2022, Sect. 3.2)). To simplify the notation, in the case where the ${\mathcal {I}}_{M}$-vestigial subnetwork is empty (${\mathcal {D}}_{{\mathcal {I}}_{M}}=\varnothing$), we write $\det (J_{{\mathcal {D}}_{{\mathcal {I}}_{M}}}) \equiv 1$. $\Diamond$

Now we need to specify the set $\Omega \subseteq {\mathbb {R}}^N$ of allowable parameter values. This set depends on the admissible vector field *F* and the type of model being considered. For instance, in a biochemical network the set $\Omega$ is the positive orthant of some ${\mathbb {R}}^N$. The subset of *non-singular parameters* of *F* on $\Omega$ is defined as

2.19 $$\begin{aligned} \Omega _J =\{{\mathcal {I}}\in \Omega : \det (J) \ne 0\}. \end{aligned}$$

where $J=(DF)_{({\tilde{X}}({\mathcal {I}}),{\mathcal {I}})}$ is the jacobian. The set $\Omega _J$ also depends on the vector field *F* and, generically, is an open dense subset of $\Omega$.

#### Lemma 2.3

For each $M = 1, \ldots , N$, we have:

2.20 $$\begin{aligned} \det \langle H_{M} \rangle = \det (J_{{\mathcal {D}}_{{\mathcal {I}}_{M}}}) \, \det \langle H_{{\mathcal {I}}_{M}} \rangle , \end{aligned}$$

Moreover, $\det (J_{{\mathcal {D}}_{{\mathcal {I}}_{M}}}) \ne 0$ over $\Omega _J$ and the irreducible factors of $\det (J_{{\mathcal {D}}_{{\mathcal {I}}_{M}}})$ never are irreducible scalar factors of $\vec {h}$.

#### Proof

In case ${\mathcal {D}}_{{\mathcal {I}}_M} = \varnothing$, the result follows from the convention that $\det (J_{{\mathcal {D}}_{{\mathcal {I}}_{M}}} ) \equiv 1$. In case ${\mathcal {D}}_{{\mathcal {I}}_{M}} \ne \varnothing$, the vestigial subnetwork is composed by nodes that are not downstream from the input nodes affected by the parameter ${\mathcal {I}}_{M}$. Hence, we can apply to ${\mathcal {G}}_{{\mathcal {I}}_{M}}$ the ‘core network’ theorem for networks with multiple input nodes and a single input parameter (Madeira and Antoneli 2022, Thm 3.2). The statement about the irreducible factors of $\det (J_{{\mathcal {D}}_{{\mathcal {I}}_{M}}})$ follows from an argument similar to the one employed in Madeira and Antoneli (2022, Prop 3.8). $\square$

Lemma 2.3 allows us to further simplify the components of $\vec {h}$, by considering the determinants $\det \langle H_{{\mathcal {I}}_{M}} \rangle$. This reduce to the situation already studied in Madeira and Antoneli (2022).

#### Definition 2.8

The *vector determinant* associated to an input–output network is the vector-valued function defined by

2.21 $$\begin{aligned} {\widehat{h}}= \big (\det \langle H_{{\mathcal {I}}_1} \rangle , \det \langle H_{{\mathcal {I}}_2} \rangle , \ldots , \det \langle H_{{\mathcal {I}}_N} \rangle \big ), \end{aligned}$$

where $\det \langle H_{{\mathcal {I}}_{M}} \rangle$, $M=1,\ldots ,N$, is the determinant of generalized homeostasis matrix of the ${\mathcal {I}}_{M}$-specialized subnetwork ${\mathcal {G}}_{{\mathcal {I}}_{M}}$. The vector-valued function ${\widehat{h}}$ can be considered as a (formal) polynomial mapping on the ‘variables’ $f_{j,x_i}$ and $f_{j,I_M}$. $\Diamond$

#### Proposition 2.4

The vector-valued functions $\nabla x_o$, $\vec {h}$ and ${\widehat{h}}$, defined on $\Omega \rightarrow {\mathbb {R}}^N$, have the same set of zeros on $\Omega _J$.

#### Proof

The first equality follows from Lemma 2.1 and the second equality follows from Lemma 2.3. $\square$

The König-Frobenius theorem (Schneider 1977; Brualdi and Cvetkoić 2009) (see also Wang et al. (2021); Madeira and Antoneli (2022)) imply that the components of the polynomial mapping ${\widehat{h}}$ can be factorized as the product of the determinants of the irreducible diagonal blocks of each $\langle H_{{\mathcal {I}}_M} \rangle$ (defined up to row and column permutations). An irreducible block *B* of some $\langle H_{{\mathcal {I}}_M} \rangle$ is called a *homeostasis block*. We can further collect the factors that are common irreducible diagonal blocks of all matrices $\langle H_{{\mathcal {I}}_M} \rangle$ and bring them to the front as scalar factors. Then we can write

2.22 $$\begin{aligned} {\widehat{h}} = \det (B_1) \cdots \det (B_k) \, \left( \prod _{j_1}\det \left( B_{{\mathcal {I}}_1}^{j_1} \right) , \ldots , \prod _{j_N}\det \left( B_{{\mathcal {I}}_N}^{j_N} \right) \right) \end{aligned}$$

Therefore, we can split the problem of classifying homeostasis types supported by ${\mathcal {G}}$ into two cases according to whether the components of ${\widehat{h}}$ have a common scalar factor or not.

#### Definition 2.9

Let ${\mathcal {G}}$ be a core multiple inputs network and consider its vector determinant ${\widehat{h}}$ as in (2.22). A homeostasis block corresponding to scalar factor $\det (B_i)$ ($i=1,\ldots ,k$) of ${\widehat{h}}$ is called a *pleiotropic homeostasis block*. The other homeostasis blocks of $\mathcal {G}$ are called *coincidental*. $\Diamond$

#### Remark 2.10

In genetics, pleiotropy refers to the phenomenon when a single locus affects multiple traits (Stearns 2010). Here, we employed the term *pleiotropic homeostasis* referring to the fact that the nullification of one single homeostasis block leads to the annulment of the whole homeostasis vector ${\widehat{h}}$. $\Diamond$

Recall that the *homeostasis types* of a single parameter input–output network ${\mathcal {G}}$ are given in terms of the factors of $h=\det (H)$ (see Wang et al. (2021); Madeira and Antoneli (2022))

2.23 $$\begin{aligned} \det (H)=\det (B_1) \cdots \det (B_k), \end{aligned}$$

where each irreducible block $B_j$ can be of three types, called *appendage*, *structural* and *counterweight*. There is only one counterweight block defined as the only irreducible block that contains all the partial derivatives of *f* with respect to the input ${\mathcal {I}}$. Infinitesimal homeostasis of type $B_j$ occurs if $\det (B_j)=0$ and $\det (B_i)\ne 0$ for all $i\ne j$. This is generic when there is only one input parameter.

Now, suppose that ${\mathcal {G}}$ has *N* inputs affecting *n* input nodes. Generically, it is expected that *N* irreducible factors of ${\widehat{h}}$ can simultaneously vanish at a fixed input value.

#### Definition 2.11

Let ${\mathcal {G}}$ be a multiple inputs core network. Let *B* be a homeostasis block of size $\ell$. We say that the *homeostasis class* of *B* is

- *Input counterweight* if *B* contains partial derivatives with respect to inputs (the simplest counterweight block is of the form $f_{\iota _m,{\mathcal {I}}_M}$),
- *Appendage* if *B* has $\ell$ self-couplings,
- *Structural* if *B* has exactly $\ell - 1$ self-couplings. $\Diamond$

It follows from the argument in (Madeira and Antoneli 2022, Sec. 3.4), applied to the specialized subnetworks ${\mathcal {G}}_{{\mathcal {I}}_M}$, that each homeostasis block of ${\mathcal {G}}$ is of one of the classes in Definition 2.11.

#### Definition 2.12

Let ${\mathcal {G}}$ be a core multiple inputs network.

- We say that *pleiotropic homeostasis* occurs when at least one pleiotropic block has vanishing determinant at some fixed input value. The pleiotropic blocks determine the *pleiotropic homeostasis types* of ${\mathcal {G}}$.
- We say that *coincidental homeostasis* occurs when a *N*-tuple of coincidental blocks $\big (B_{{\mathcal {I}}_1}^{j_1},\ldots , B_{{\mathcal {I}}_N}^{j_N}\big )$ has simultaneously vanishing determinants at some fixed input value. The *N*-tuples of coincidental blocks determine the *coincidental homeostasis types* of ${\mathcal {G}}$. $\Diamond$

#### Definition 2.13

Let ${\mathcal {G}}$ be a core multiple inputs network and *B* be a homeostasis block. The *homeostasis subnetwork* ${\mathcal {K}}_B$ associated to *B* is defined as follows. The nodes of ${\mathcal {K}}_B$ are the nodes $\sigma$ and $\rho$ of ${\mathcal {G}}$ such that $f_{\sigma ,x_{\rho }}$ is a non-zero entry of *B*. The arrows of ${\mathcal {K}}_B$ are the arrows $\sigma \rightarrow \rho$ of ${\mathcal {G}}$ such that $\sigma ,\rho \in {\mathcal {K}}_B$ with $\sigma \ne \rho$. $\Diamond$

### Pleiotropic homeostasis types

In this section we classify the pleiotropic sub-blocks of a multiple inputs core network.

#### Proposition 2.5

Let ${\mathcal {G}}$ be a multiple inputs core network and *B* be a pleiotropic block of ${\mathcal {G}}$.

(i) Then *B* is either appendage or structural.
(ii) More precisely, *B* is an appendage (respect. structural) block if and only if it is $\langle H_{{\mathcal {I}}_M} \rangle$-appendage (respect. $\langle H_{{\mathcal {I}}_M} \rangle$-structural) block, for all $M = 1, \ldots , N$.

#### Proof

(i) From the results of Wang et al. (2021), Madeira and Antoneli (2022), we see that, for each $M = 1, \ldots , N$, *B* can be classified with respect to the specialized subnetwork ${\mathcal {G}}_{{\mathcal {I}}_{M}}$ associated to the input ${\mathcal {I}}_{M}$ as an $\langle H_{{\mathcal {I}}_M} \rangle$-input counterweight, an $\langle H_{{\mathcal {I}}_M} \rangle$-structural or an $\langle H_{{\mathcal {I}}_M} \rangle$-appendage block. As the derivatives $f_{\iota _{j}, {\mathcal {I}}_{M}}$ would appear in the expression of $\det (B)$ if it was a $\langle H_{{\mathcal {I}}_M} \rangle$-input counterweight block, we conclude that $\det (B)$ must be either an $\langle H_{{\mathcal {I}}_M} \rangle$-structural or an $\langle H_{{\mathcal {I}}_M} \rangle$-appendage block, for all $M = 1, \ldots , N$.

(ii) This follows immediately from the fact that the classification is based on the number of self-coupling entries. $\square$

In Remark 2.4 we observed that when the network ${\mathcal {G}}$ has a single parameter the theory developed here reduces to the situation considered in Madeira and Antoneli (2022). The next result shows that when the multiple inuts network ${\mathcal {G}}$ has a single input node then the theory essentially reduces to the case where there is only one input parameter, considered in Wang et al. (2021). This is an extreme case where only pleiotropic homeostasis occurs.

#### Proposition 2.6

Suppose the core multiple inputs network ${\mathcal {G}}$ has only one input node $\iota$ and multiple inputs ${\mathcal {I}}_{1}, \ldots , {\mathcal {I}}_{N}$. Then, we have:

$$\begin{aligned} \nabla x_{o} = \frac{\det (H)}{\det (J)} \, \left( f_{\iota , {\mathcal {I}}_{1}}, \ldots , f_{\iota , {\mathcal {I}}_{N}} \right) \end{aligned}$$

where $\det (H)$ is the homeostasis determinant of the network ${\mathcal {G}}$, as a polynomial function of the partial derivatives $f_{j,x_i}$. In particular, Condition (2.8) implies that infinitesimal homeostasis occurs if and only if $\det (H) = 0$.

#### Proof

This is a consequence of Lemma 2.1 and of Eq. (2.16) for $\det \langle H_{M} \rangle$ when ${\mathcal {G}}$ has a single input node. $\square$

Therefore, Proposition 2.6 implies that the classification of homeostasis types of a multiple inputs network with a single input node is exactly the same as that of a single input parameter single input node network. However, it is not true that we expect to see the same ‘homeostasis phenomena’ in both networks. In order to see this, suppose that ${\mathcal {G}}$ has a single input node, but multiple inputs $({\mathcal {I}}_1,\ldots ,{\mathcal {I}}_N)$ affecting the input node. Consequently, the function *h*, which is the same polynomial on the partial derivatives $f_{j,x_i}$, becomes a multivariate function when evaluated at ${\tilde{X}}({\mathcal {I}}_1,\ldots ,{\mathcal {I}}_N)$. Generically, it is expected that *N* irreducible factors of (2.23) can simultaneously vanish at a fixed value $({\mathcal {I}}_1^0,\ldots ,{\mathcal {I}}_N^0)$.

Returning to the general case, the next result gives a complete topological classification of the homeostasis subnetworks corresponding to the pleiotropic homeostasis types.

#### Theorem 2.7

Let ${\mathcal {G}}$ be a multiple inputs core network, *B* be a pleiotropic block of ${\mathcal {G}}$ and ${\mathcal {K}}_B$ be the corresponding homeostasis subnetwork to *B* in ${\mathcal {G}}$.

(i) Then ${\mathcal {K}}_B$ is either an appendage or structural subnetwork of all ${\mathcal {I}}_M$-specialized subnetworks ${\mathcal {G}}_{{\mathcal {I}}_M}$.
(ii) More precisely, ${\mathcal {K}}_B$ is an appendage (respect. structural) subnetwork if and only if it is a $\langle H_{{\mathcal {I}}_M} \rangle$-appendage (respect. $\langle H_{{\mathcal {I}}_M} \rangle$-structural) subnetwork, for some (and hence for all) $M = 1, \ldots , N$.

#### Proof

The result follows from Theorems  4.3 and 4.4 for the appendage case and Theorems 4.8 and 4.9 for the structural case. See Sect. 4.2 for precise characterization of each type of subnetwork. $\square$

### Coincidental homeostasis types

The occurrence of coincidental homeostasis reflects the fact that the mechanism leading to homeostasis is not the same with respect to all the inputs. The coincidental homeostasis types are given by all the possible combinations of coincidental blocks. A coincidental type can be of structural class, appendage class or input counterweight class. Thus, depending on the network, a coincidental type can have the form of a mix of these three classes.

In the simplest case the determinant of a coincidental block can be an entry of some $\langle H_{{\mathcal {I}}_M} \rangle$ of the form $f_{x_{\iota _m},{\mathcal {I}}_M}$. Since, by assumption, these entries cannot vanish it may happen that some coincidental types do not yield infinitesimal homeostasis.

On one hand, as shown in Proposition 2.6, networks that have only one input node, only support pleiotropic homeostasis. On the other hand, it is easy to find networks that support only coincidental homeostasis (see Sect. 2.9).

Still, one may wonder whether there is a multiple inputs core network that do not support either pleiotroic nor coincidental homeostasis, that is, it does not support infinitesimal homeostasis. The next proposition shows that this cannot happen, namely, any multiple inputs core network always support at least one type of homeostasis.

#### Proposition 2.8

A multiple inputs core network ${\mathcal {G}}$ always supports infinitesimal homeostasis.

#### Proof

In order to prove the proposition, consider all input nodes $\iota _{m}$, such there is an $\iota _{m}$-simple node $\sigma$ ($\sigma \ne \iota _{m}$) that receives an arrow from $\iota _{m}$ and such that $f_{\sigma , x_{\iota _{m}}} = 0$ (Haldane homeostasis). Let ${\mathcal {G}}_{m}$ be the core subnetwork between the input node $\iota _{m}$ and output node *o* (Madeira and Antoneli 2022, Def. 2.13). By definition, ${\mathcal {G}}_{m}$ is a single input–output network. Then, by the characterization of structural homeostasis in networks with single one input node (see Wang et al. 2021), the homeostasis determinant of ${\mathcal {G}}_{m}$ vanish, i.e., if $H^{c}_{\iota _{m}}$ is the homeostasis matrix of ${\mathcal {G}}_{m}$, then $\det H^{c}_{\iota _{m}} = 0$. This fact together with (Madeira and Antoneli 2022, Eq. 3.39) implies that $\det \langle H_{{\mathcal {I}}_{M}} \rangle = 0$, for all $M = 1, \ldots , N$. To conclude the argument, we claim that this construction does not force $\det (J)=0$, generically. This follows from that fact that one of the terms that appear in the expression of the Jacobian determinant is the product of all the self-couplings of nodes of ${\mathcal {G}}$. As the construction above does not assume that the self-couplings to be equal to zero, the claim holds. $\square$

Finally, we state below a sufficient condition for a core multiple inputs network to support coincidental homeostasis. The network of Fig. 4b shows that the condition given below is sufficient, but not necessary, for a core multiple inputs network to support coincidental homeostasis.

#### Proposition 2.9

Let ${\mathcal {G}}$ be a multiple inputs core network. If none of the input nodes of ${\mathcal {G}}$ is an absolutely super-simple node (see Definition 4.1), then ${\mathcal {G}}$ supports at least one coincidental homeostasis type.

#### Proof

Note that an input node is an absolutely super-simple node if and only if it is the first absolutely super-simple node of the network. Hence, the hypothesis that none of the input nodes of ${\mathcal {G}}$ is an absolutely super-simple node is equivalent to: the first absolutely super-simple node of ${\mathcal {G}}$ is not an input node. First, suppose that pleiotropic homeostasis does not occurs in ${\mathcal {G}}$. Then by Proposition 2.8 it follows that coincidental homeostasis must occur in ${\mathcal {G}}$. Now suppose that pleiotropic homeostasis does occur in ${\mathcal {G}}$. Clearly, as the input nodes are not absolutely appendage, by Theorem 2.7, they do not belong to a pleiotropic-appendage subnetwork. In addition, none of the input nodes belong to any absolutely super-simple structural subnetwork, as the input nodes are not absolutely super-simple and cannot be between two absolutely super-simple nodes (this is a consequence of Madeira and Antoneli (2022, Lem 3.15)). Hence, by Theorem 2.7, none of the input nodes belong to a pleiotropic-structural subnetwork. Therefore, each input node belong to a counterweight subnetwork and thus coincidental homeostasis must occur. $\square$

#### Remark 2.14

As previously explained, Proposition 2.9 does not provide us with a necessary condition for the occurrence of coincidental homeostasis. It is then a open problem to determine necessary and sufficient conditions for the occurrence of coincidental homeostasis. As it will be exemplified in Sect. 2.9, the interaction between the different subnetworks makes this a non-trivial problem. $\Diamond$

### Algorithm to determine all homeostasis types

Using the results obtained here, together with Wang et al. (2021); Madeira and Antoneli (2022), one can write down a general algorithm to find all homeostasis types of a given multiple inputs core network ${\mathcal {G}}$.

*Step 1*: For each parameter ${\mathcal {I}}_M$ with $M=1,\ldots ,N$ determine the ${\mathcal {I}}_M$-specialized subnetwork ${\mathcal {G}}_{{\mathcal {I}}_M}$ as in Definition 2.6.
*Step 2*: Since each ${\mathcal {G}}_{{\mathcal {I}}_M}$ is a single parameter multiple input–output network one can apply the algorithm of (Madeira and Antoneli 2022, Sect. 2.6) to determine all the homeostasis subnetworks ${\mathcal {K}}$ of each ${\mathcal {G}}_{{\mathcal {I}}_M}$.
*Step 3*: Determine the homeostasis subnetworks that are common to all ${\mathcal {G}}_{{\mathcal {I}}_M}$. The output of this step is the list of pleiotropic homeostasis types.
*Step 4*: Determine the *N*-tuples formed by combinations of the remaining homeostasis subnetworks. The output of this step is the list of coincidental homeostasis types.

### Some simple examples

In this section we present some examples of small networks exhibiting an astonishing array of phenomena that can arise in the multiparameter setting.

For instance, there are networks that do support both pleiotropic and coincidental homeostasis (e.g. Example 2.16) and networks that support only one of each type: Example 2.15 supports only pleiotropic homeostasis. Whereas Example (...) supports only coincidental homeostasis.

Moreover, we will see that when there is a *proper coincidental type* (i.e. all determinants can vanish) non-trivial interaction between the homeostasis subnetworks can occur.

Let us start with the minimal non-trivial examples. There are some constraints to get non-trivial networks:

- There must be at least two inputs.
- There must be at least two input nodes (see Proposition 2.6).
- The output node is distinct from the input nodes (see Remark 2.2).

**Fig. 3:**
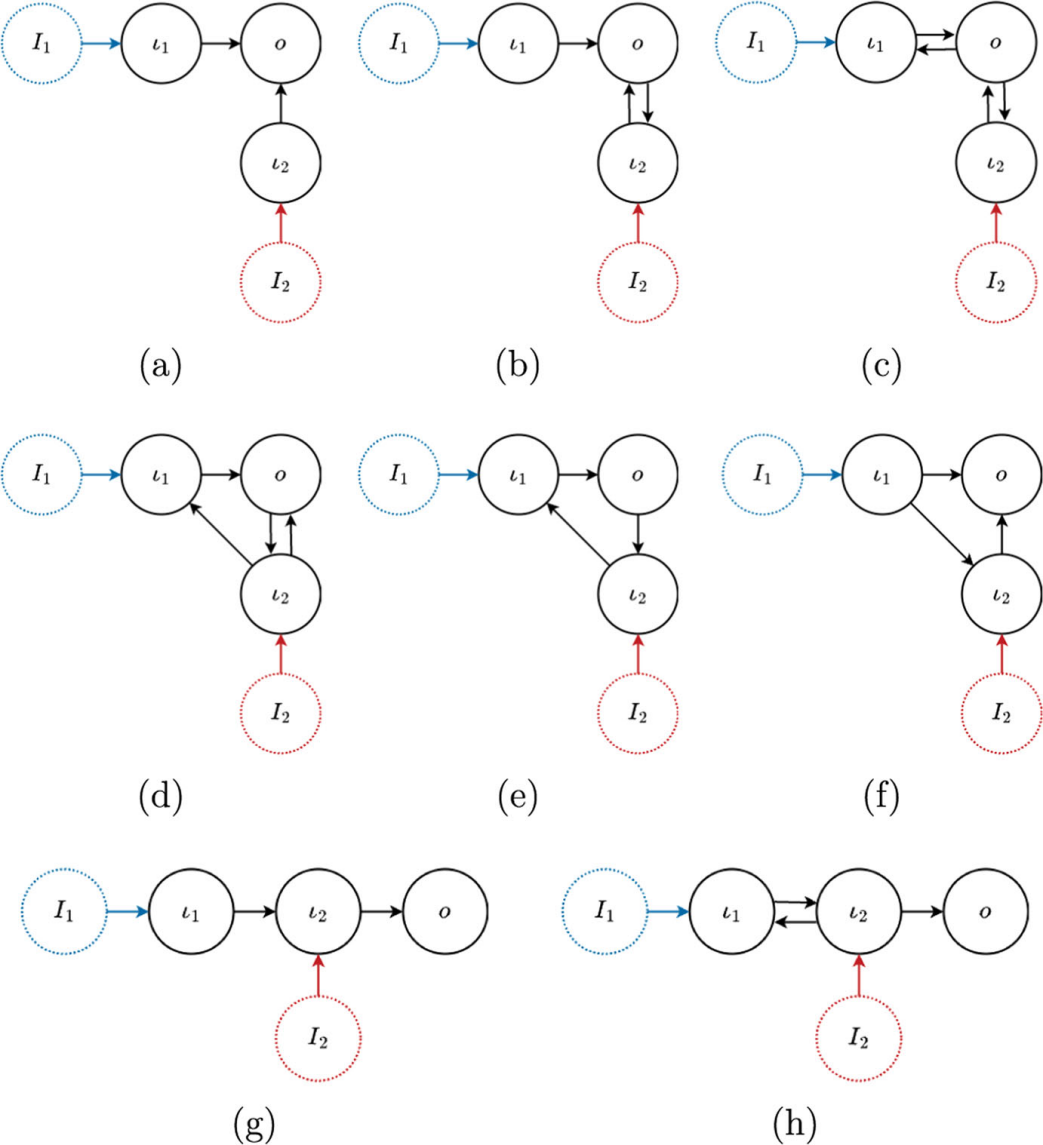
Three-node core networks with one input per input node

Therefore, the network must have at least three nodes, two input nodes $\iota _1$, $\iota _2$ and the output node *o*. In addition, let us make the simplifying assumption that each input parameter affects exactly one input node. Granted these conditions, it is not difficult to show that, up to core-equivalence and input-relabeling, there are exactly 8 networks, shown in Fig. 3. Two core networks are *core-equivalent* if they have identical vector determinants, up to permutation (cf. Wang et al. 2021, Def. 1.9).

#### Example 2.15

Consider the eight 3-node core networks shown in Fig. 3. We begin by determining the specialized subnetworks. Since the networks have one input per input node the specialized subnetwork is a single parameter single input 3-node network. Then we can use the results from Golubitsky and Wang (2020) to write the vector determinant. We also note that, by definition, $f_{\iota _{1},{\mathcal {I}}_{1}}$ and $f_{\iota _{2},{\mathcal {I}}_{2}}$ cannot vanish.

**Network (a).**  In this example we have

$$\begin{aligned} {{\mathcal {I}}_1 \rightarrow \,} \iota _1 \rightarrow o \qquad \qquad \qquad \qquad {{\mathcal {I}}_2 \rightarrow \;} \iota _2 \rightarrow o \end{aligned}$$

Therefore, the vector determinant is given by

$$\begin{aligned} {\widehat{h}} = \big (f_{\iota _{1},{\mathcal {I}}_{1}} f_{o,x_{\iota _1}}, f_{\iota _{2},{\mathcal {I}}_{2}} f_{o,x_{\iota _{2}}} \big ). \end{aligned}$$

Hence, the network only supports coincidental homeostasis, given by the simultaneous vanishing of the irreducible (structural) factors $f_{o,x_{\iota _1}}$ and $f_{o,x_{\iota _{2}}}$.

**Network (b).**  In this example we have

$$\begin{aligned} {{\mathcal {I}}_1 \rightarrow \;} \iota _1 \rightarrow \overset{\overset{{\displaystyle \iota _2}}{\uparrow \downarrow }}{o} \qquad \qquad \qquad \qquad {{\mathcal {I}}_2 \rightarrow \;} \iota _2 \rightarrow o \end{aligned}$$

In the second specialized network we omitted the arrow $o \rightarrow \iota _2$, since it does not affect the vector determinant. Therefore, the vector determinant is given by

$$\begin{aligned} {\widehat{h}} = \big (f_{\iota _{1},{\mathcal {I}}_{1}} f_{o,x_{\iota _{1}}} f_{\iota _2,x_{\iota _2}}, f_{\iota _{2},{\mathcal {I}}_{2}} f_{o,x_{\iota _2}}\big ). \end{aligned}$$

Hence, the network does not support pleiotropic homeostasis. There are two possibilities for the occurrence of coincidental homeostasis

1. $f_{o,x_{\iota _1}}$ (structural) and $f_{o,x_{\iota _2}}$ (structural),
2. $f_{\iota _2,x_{\iota _2}}$ (appendage) and $f_{o,x_{\iota _2}}$ (structural).

Now, the jacobian determinant of the network is

$$\begin{aligned} \det (J) = f_{\iota _1,x_{\iota _1}} \, \big (f_{\iota _2,x_{\iota _2}} f_{o,x_o} - f_{\iota _2,x_o} f_{o,x_{\iota _2}} \big ). \end{aligned}$$

Hence, the occurrence of homeostasis in case (2) necessarily forces $\det (J)=0$ (see Remark 2.17). Therefore, coincidental homeostasis only can occur in case (1).

**Network (c).**  In this example we have

$$\begin{aligned} {{\mathcal {I}}_1 \rightarrow \;} \iota _1 \rightarrow \overset{\overset{{\displaystyle \iota _2}}{\uparrow \downarrow }}{o} \qquad \qquad \qquad \qquad {{\mathcal {I}}_2 \rightarrow \;} \iota _2 \rightarrow \overset{\overset{{\displaystyle \iota _1}}{\uparrow \downarrow }}{o} \end{aligned}$$

Therefore, the vector determinant is given by

$$\begin{aligned} {\widehat{h}} = \big (f_{\iota _{1},{\mathcal {I}}_{1}} f_{o,x_{\iota _{1}}} f_{\iota _2,x_{\iota _2}}, f_{\iota _{2},{\mathcal {I}}_{2}} f_{o,x_{\iota _{2}}} f_{\iota _1,x_{\iota _1}} \big ). \end{aligned}$$

Now, the jacobian determinant of the network is

$$\begin{aligned} \det (J) = f_{\iota _1,x_{\iota _1}} f_{\iota _2,x_{\iota _2}} f_{o,x_o} - f_{\iota _1,x_o} f_{\iota _2,x_{\iota _2}} f_{o,x_{\iota _1}} - f_{\iota _2,x_o} f_{\iota _1,x_{\iota _1}} f_{o,x_{\iota _2}} \end{aligned}$$

Hence, the network only supports coincidental homeostasis. Moreover, from the 4 possible combinations of factors of ${\widehat{h}}$ the only that does not force $\det (J)=0$ is given by $f_{o,x_{\iota _1}}$ and $f_{o,x_{\iota _2}}$ (see Remark 2.17).

**Network (d).**  In this example we have

$$\begin{aligned} {{\mathcal {I}}_1 \rightarrow \;} \iota _1\overset{\overset{{\displaystyle \iota _2}}{\nearrow \quad \searrow }}{\rightarrow } o \qquad \qquad \qquad \qquad {{\mathcal {I}}_2 \rightarrow \;} \iota _2\overset{\overset{{\displaystyle \iota _1}}{\nearrow \quad \searrow }}{\rightarrow } o \end{aligned}$$

In both specialized networks we omitted the arrow $o\rightarrow \iota _2$ since it does not affect the vector determinant. Therefore, the vector determinant is given by

$$\begin{aligned} {\widehat{h}} = \big (f_{\iota _{1},{\mathcal {I}}_{1}} (f_{\iota _2,x_{\iota _1}} f_{o,x_{\iota _2}}-f_{o,x_{\iota _1}} f_{\iota _2,x_{\iota _2}}), f_{\iota _{2},{\mathcal {I}}_{2}} (f_{\iota _1,x_{\iota _2}} f_{o,x_{\iota _1}}-f_{o,x_{\iota _2}} f_{\iota _1,x_{\iota _1}}) \big ). \end{aligned}$$

Hence, the network only supports coincidental homeostasis, given by the simultaneous vanishing of the two irreducible (structural) factors.

**Network (e).**  In this example we have

$$\begin{aligned} {{\mathcal {I}}_1 \rightarrow \;} \iota _1 \overset{\overset{{\displaystyle \iota _2}}{\swarrow \quad \nwarrow }}{\rightarrow } o \qquad \qquad \qquad \qquad {{\mathcal {I}}_2 \rightarrow \;} \iota _2 \rightarrow \iota _1 \rightarrow o \end{aligned}$$

In the second specialized network we omitted the arrow $o \rightarrow \iota _2$, since it does not affect the vector determinant. Therefore, the vector determinant is given by

$$\begin{aligned} {\widehat{h}} = f_{o,x_{\iota _1}} \big (f_{\iota _{1},{\mathcal {I}}_{1}} f_{\iota _2,x_{\iota _2}}, f_{\iota _{2},{\mathcal {I}}_{2}} f_{\iota _1,x_{\iota _2}} \big ). \end{aligned}$$

Hence, the network does support pleiotropic homeostasis. In order for coincidental homeostasis to occur, both irreducible factors below must vanish simultaneously

$$\begin{aligned} f_{\iota _2,x_{\iota _2}} \qquad \text {and}\qquad f_{\iota _1,x_{\iota _2}} \end{aligned}$$

Now the jacobian determinant of the network is

$$\begin{aligned} \det (J) = f_{\iota _1,x_{\iota _1}} f_{\iota _2,x_{\iota _2}} f_{o,x_{o}} + f_{\iota _1,x_{\iota _2}} f_{\iota _2,x_{o}} f_{o,x_{\iota _1}} \end{aligned}$$

Thus, the vanishing of the two factors force $\det (J)$ to vanish and so coincidental homeostasis can not occur in this network (see Remark 2.17).

**Network (f).**  In this example we have

$$\begin{aligned} {{\mathcal {I}}_1 \rightarrow \;} \iota _1 \overset{\overset{{\displaystyle \iota _2}}{\nearrow \quad \searrow }}{\rightarrow } o \qquad \qquad \qquad \qquad {{\mathcal {I}}_2 \rightarrow \;} \iota _2 \rightarrow o \end{aligned}$$

Therefore, the vector determinant is given by

$$\begin{aligned} {\widehat{h}} = \big (f_{\iota _{1},{\mathcal {I}}_{1}} (f_{\iota _{2},x_{\iota _{1}}} f_{o,x_{\iota _2}}-f_{o,x_{\iota _1}} f_{\iota _2,x_{\iota _2}}), f_{\iota _{2}, {\mathcal {I}}_{2}} f_{o,x_{\iota _{2}}} \big ). \end{aligned}$$

Hence, the network does not support pleiotropic homeostasis. In order for coincidental homeostasis to occur, both irreducible factors below must vanish simultaneously

$$\begin{aligned} f_{\iota _{2},x_{\iota _{1}}} f_{o,x_{\iota _2}}-f_{o,x_{\iota _1}} f_{\iota _2,x_{\iota _2}} \qquad \text {and}\qquad f_{o,x_{\iota _2}}. \end{aligned}$$

Note that both factors are of structural homeostasis type. The vanishing of $f_{o,x_{\iota _2}}$ reduces the first factor to $f_{o,x_{\iota _1}} f_{\iota _2,x_{\iota _2}}$. The jacobian determinant of the network is

$$\begin{aligned} \det (J) = f_{\iota _1,x_{\iota _1}} f_{\iota _2,x_{\iota _2}} f_{o,x_{o}} \end{aligned}$$

Hence, the condition $f_{\iota _2,x_{\iota _2}}=0$ forces $\det (J)=0$, whereas $f_{o,x_{\iota _1}}=0$ does not (see Remark 2.17). Therefore, the vanishing of the factor $f_{o,x_{\iota _1}}0$ is the only possibility for the occurrence of coincidental homeostasis.

**Network (g).**  In this example we have

$$\begin{aligned} {{\mathcal {I}}_1 \rightarrow \;} \iota _1 \rightarrow \iota _2 \rightarrow o \qquad \qquad \qquad \qquad {{\mathcal {I}}_2 \rightarrow \;} \iota _2 \rightarrow o \end{aligned}$$

Therefore, the vector determinant is given by

$$\begin{aligned} {\widehat{h}} = f_{o, x_{\iota _{2}}} \, \big (f_{\iota _{1}, {\mathcal {I}}_{1}} f_{\iota _{2}, x_{\iota _{1}}}, f_{\iota _{2}, {\mathcal {I}}_{2}} \big ). \end{aligned}$$

Hence, the network does not support coincidental homeostasis. In order for pleiotropic homeostasis to occur the irreducible (structural) factor $f_{o,x_{\iota _{2}}}$ must vanish. Here, the obstruction to the occurrence of coincidental homeostasis is due to the fact that the second component of ${\hat{h}}$ consists only of the non-vanishing factor $f_{\iota _{2},{\mathcal {I}}_{2}}$.

**Network (h).**  In this example we have

$$\begin{aligned} {{\mathcal {I}}_1 \rightarrow \;} \iota _1 \rightarrow \iota _2 \rightarrow o \qquad \qquad \qquad \qquad {{\mathcal {I}}_2 \rightarrow \;} \overset{\overset{{\displaystyle \iota _1}}{\uparrow \downarrow }}{\iota _2} \rightarrow o \end{aligned}$$

In the first specialized network we omitted the arrow $\iota _2 \rightarrow \iota _1$, since it does not affect the vector determinant. Therefore, the vector determinant is given by

$$\begin{aligned} {\widehat{h}} = f_{o, x_{\iota _{2}}} \, \big (f_{\iota _{1}, {\mathcal {I}}_{1}} f_{\iota _{2}, x_{\iota _{1}}}, f_{\iota _{2}, {\mathcal {I}}_{2}} f_{\iota _1,x_{\iota _1}} \big ). \end{aligned}$$

Hence, pleiotropic homeostasis occurs when the irreducible (structural) factor $f_{o,x_{\iota _{2}}}$ vanishes. Coincidental homeostasis is given by the simultaneous vanishing of the irreducible factors $f_{\iota _2,x_{\iota _1}}$ and $f_{\iota _1,x_{\iota _1}}$. $\Diamond$

**Fig. 4:**
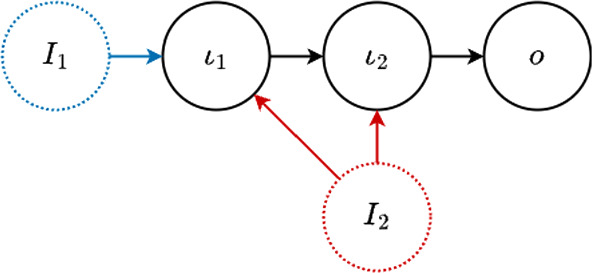
Three-node core network with input affecting both input nodes

Next, we consider an example obtained from the network shown in Fig. 3g by adding the influence from input ${\mathcal {I}}_2$ on node $\iota _1$. The main difference from the networks in Example 2.15 is the appearance of an input counterweight factor. This illustrates the effect of having more than one input node affected by the same input (see Fig. 4). Note that this is core equivalent to adding the influence from input ${\mathcal {I}}_2$ on node $\iota _1$ on the network shown in Fig. 3h.

#### Example 2.16

Consider the 2-parameter 3-node core network shown in Fig. 4. The specialized subnetworks are:

$$\begin{aligned} {{\mathcal {I}}_1 \rightarrow \;} \iota _1 \rightarrow \iota _2 \rightarrow o \qquad \qquad \qquad \qquad \iota _1 \underset{\underset{{\displaystyle { {\mathcal {I}}_2}}}{\nwarrow \quad \nearrow }}{\rightarrow } \iota _2 \rightarrow o \end{aligned}$$

Therefore, the vector determinant is given by

$$\begin{aligned} {\widehat{h}} = f_{o, x_{\iota _{2}}} \, \big (f_{\iota _{1}, {\mathcal {I}}_{1}} f_{\iota _{2}, x_{\iota _{1}}}, f_{\iota _{1}, {\mathcal {I}}_{2}} f_{\iota _{2}, x_{\iota _{1}}} - f_{\iota _{2}, {\mathcal {I}}_{2}} f_{\iota _{1}, x_{\iota _{1}}} \big ). \end{aligned}$$

Pleiotropic homeostasis can occur by the vanishing of the irreducible (structural) factor $f_{o,x_{\iota _{2}}}$. In order for coincidental homeostasis to occur both irreducible factors (the first is structural and the second is input counterweight) below must vanish simultaneously

$$\begin{aligned} \det \left( B_{{\mathcal {I}}_1}^2 \right) = f_{\iota _{2}, x_{\iota _{1}}} \qquad \text {and}\qquad \det \left( B_{{\mathcal {I}}_2}^1 \right) = f_{\iota _{1}, {\mathcal {I}}_{2}} f_{\iota _{2}, x_{\iota _{1}}} - f_{\iota _{2}, {\mathcal {I}}_{2}} f_{\iota _{1}, x_{\iota _{1}}}. \end{aligned}$$

Occurrence of coincidental homeostasis requires the simultaneous vanishing of $f_{\iota _{2}, x_{\iota _1}}$ and $f_{\iota _{1},x_{\iota _{1}}}$. Now, the jacobian determinant of the network is

$$\begin{aligned} \det (J) = f_{o, x_{o}} \left( f_{\iota _{1}, x_{\iota _{1}}}f_{\iota _{2}, x_{\iota _{2}}} - f_{\iota _{1}, x_{2}}f_{\iota _{2}, x_{1}} \right) . \end{aligned}$$

Hence the vanishing of the two factors above forces $\det (J)=0$. Thus, coincidental homeostasis cannot occur in this network (see Remark 2.17). $\Diamond$

#### Remark 2.17

In three of the eight networks of Example 2.15, networks (b), (e), (f), and the network of Example 2.16, we faced a situation where the vanishing of certain factors, that could cause coincidental homeostasis, forced the vanishing of the jacobian determinant of the network. In other words, the homeostasis point occurs at the same value of the input parameters that makes the family of equilibria to undergo a steady-state bifurcation. Strictly speaking, this means that the situation mentioned above cannot be considered as a ‘proper’ infinitesimal homeostasis and thus we have excluded these cases. Indeed, the definition of infinitesimal homeostasis (Definition 2.1) excludes the simultaneous occurrence of homeostasis and steady-state bifurcation. However, if one considers extending the definition of homeostasis to include such cases (see Duncan et al. (2018); Duncan and Golubitsky (2019) for some advances in this direction) then one may get a much richer variety of phenomena. $\Diamond$

## Application of the theory to a model of calcium and phosphate homeostasis

In this section, we shall apply our theory to a ‘real biological system’, a mathematical model for the metabolic regulation of calcium and phosphate proposed by Granjon et al. (2017). Our aim here is to exemplify an application of the results of this paper to mathematical model from the literature, rather than exploit the details of calcium metabolism under physiological and pathological conditions.

Calcium is an essential metal ion that takes part in many signaling pathways and biochemical processes, including bone metabolism. Hence, its extracellular concentration must be tightly regulated (Blaine et al. 2015). Importantly, the regulation of extracellular calcium concentration is coupled to phosphate homeostasis (Blaine et al. 2015), which is an anion essential to human body. An explanation of the many pathways involved in calcium and phosphate metabolism is beyond the scope of this paper, and the interested reader is referred to Blaine et al. (2015) and Melmed et al. (2015, Ch 29).

**Fig. 5:**
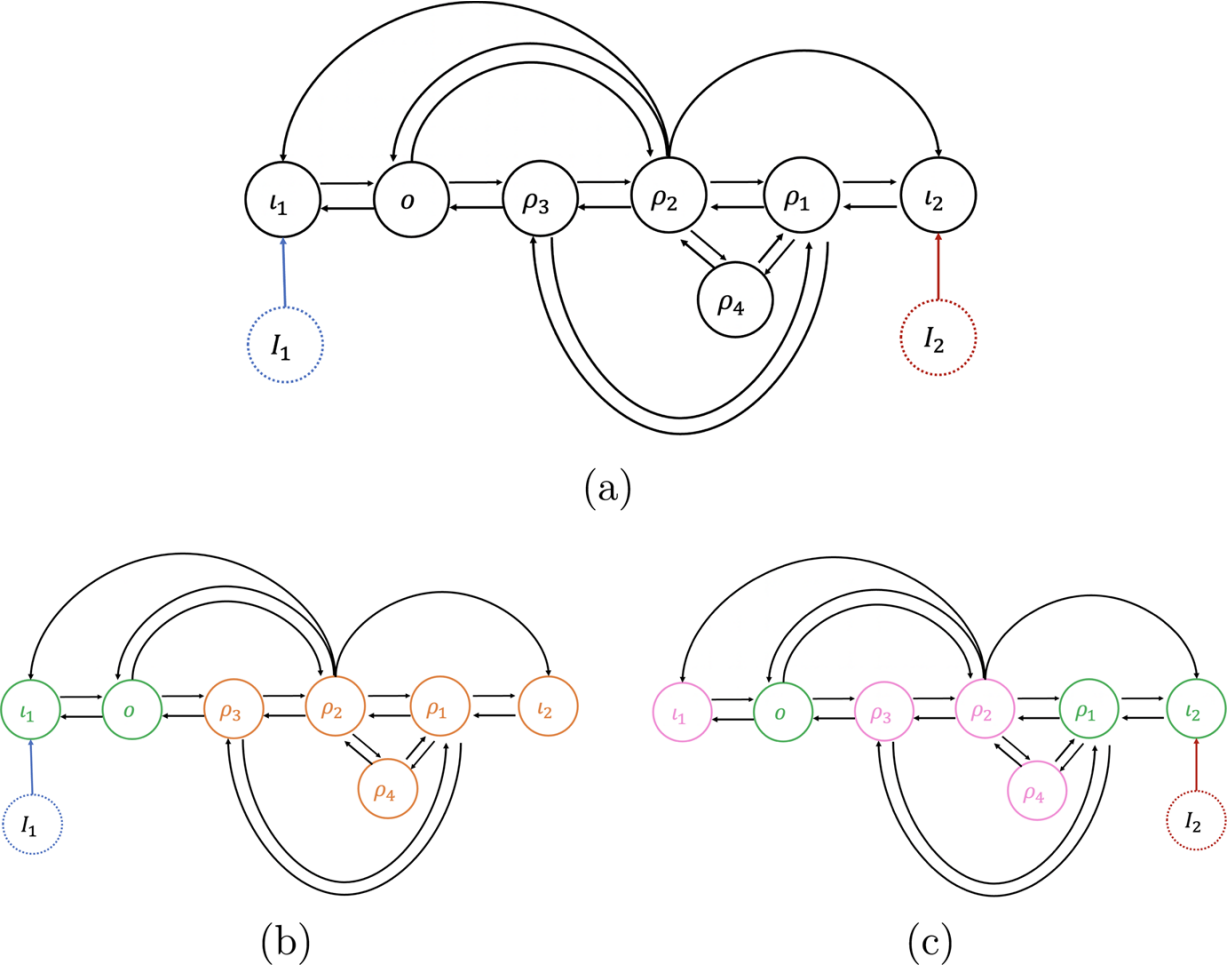
The network associated to the dynamical system (3.1) and its associated specialized subnetworks. **a** The core network associated to (3.1). From a modeling perspective, ${\mathcal {I}}_{1}$ represents the calcium intake, ${\mathcal {I}}_{2}$ the phosphate intake, $x_{\iota _{1}}$ the intestinal concentration of free calcium, $x_{\iota _{2}}$ the intestinal concentration of free phosphate, $x_{o}$ the extracellular concentration of calcium, $x_{\rho _{1}}$ the extracellular phosphate concentration, $x_{\rho _{2}}$ the concentration of calcitriol, $x_{\rho _{3}}$ the PTH concentration and $x_{\rho _{4}}$ the FGF23 concentration. **b** The ${\mathcal {I}}_{1}$-specialized subnetwork ${\mathcal {G}}_{{\mathcal {I}}_{1}}$. The ${\mathcal {I}}_{1}$-absolutely super-simple nodes are in green and the appendage nodes in orange. **c** The ${\mathcal {I}}_{2}$-specialized subnetwork ${\mathcal {G}}_{{\mathcal {I}}_{2}}$. The ${\mathcal {I}}_{2}$-absolutely super-simple nodes are in green and the other ${\mathcal {I}}_{2}$-absolutely simple nodes in pink. Since ${\mathcal {G}}_{{\mathcal {I}}_{2}}$ has no appendage nodes, it does not support appendage homeostasis. Consequently, the system does not support pleiotropic-appendage homeostasis. Moreover, since the absolutely super-simple nodes of ${\mathcal {G}}_{{\mathcal {I}}_{1}}$ and ${\mathcal {G}}_{{\mathcal {I}}_{2}}$ are different from each other (with the exception of *o*), by Theorem 4.8, the network does not support pleiotropic-structural homeostasis. Consequently, the network supports only coincidental homeostasis. All the nodes belong to both ${\mathcal {I}}_{M}$-specialized subnetworks ${\mathcal {G}}_{{\mathcal {I}}_{M}}$, for $M = 1$ and $M = 2$

The model used here is adapted from Granjon et al. (2017) and can be described by a network with 7 nodes (two input nodes, one output nodes and four regulatory nodes) and two inputs (see Fig. 5). The two inputs represent (1) the calcium intake $({\mathcal {I}}_{1})$ and (2) the phosphate intake $({\mathcal {I}}_{2})$. This is a quite natural choice, since one can assume that the intake of these ions depend on the availability of food, which can be reasonably variable. These inputs directly affect, respectively, the dynamics of the intestinal concentration of free calcium $(x_{\iota _{1}})$ and of free phosphate $(x_{\iota _{2}})$. Hence, these concentrations correspond to the two input nodes. The output node corresponds to the extracellular concentration of calcium $x_{o}$. Since calcium and phosphate metabolism is regulated by a complex network of hormones and signaling molecules, we include here the components that were highlighted by Granjon et al. (2017). The four regulatory nodes are (1) the extracellular phosphate concentration $x_{\rho _{1}}$, (2) the concentration of calcitriol, i.e. the active form of vitamin D, $x_{\rho _{2}}$, (3) the PTH concentration $x_{\rho _{3}}$ and (4) the FGF23 concentration $x_{\rho _{4}}$. Calcitriol, PTH and FGF23 are hormones that regulate bone metabolism, kidney reabsorption and intestinal absorption of calcium and phosphate, respectively (Melmed et al. 2015, Ch 29).

Following the abstract formulation introduced in Sect. 2.3, we can describe this dynamical system by the following system of ODEs

3.1 $$\begin{aligned} \begin{aligned} {\dot{x}}_{\iota _{1}}&= f_{\iota _{1}}( \iota _{1}, \rho _2, o,{\mathcal {I}}_{1}) \\ {\dot{x}}_{\iota _{2}}&= f_{\iota _{2}}( \iota _{2}, \rho _{1}, \rho _{2},{\mathcal {I}}_{2}) \\ {\dot{x}}_{\rho _{1}}&= f_{\rho _{1}}(\iota _{2}, \rho _{1}, \rho _{2}, \rho _{3}, \rho _{4}, o) \\ {\dot{x}}_{\rho _{2}}&= f_{\rho _{2}}(\rho _{1}, \rho _{2}, \rho _{3}, \rho _{4}, o) \\ {\dot{x}}_{\rho _{3}}&= f_{\rho _{3}}(\rho _{1}, \rho _{2}, \rho _{3}, o) \\ {\dot{x}}_{\rho _{4}}&= f_{\rho _{4}}(\rho _{1}, \rho _{2}, \rho _{4}) \\ {\dot{x}}_{o}&= f_{o}(\iota _{1}, \rho _{1}, \rho _{2}, \rho _{3}, o) \end{aligned} \end{aligned}$$

Since we our aim will be to describe the possible types of homeostasis supported by this system rather then to analyze the specific values that the dynamical system can assume, the abstract formulation of the system as given in (3.1) will be enough to our purposes. We refer the reader interested in the precise formulation of the system to Granjon et al. (2017). The multiple-input single-output network associated to the dynamical system above is given by Fig. 5.

The homeostasis determinants with respect to the inputs ${\mathcal {I}}_{1}$ and ${\mathcal {I}}_{2}$, respectively, are given by

3.2 $$\begin{aligned} \det \langle H_{1} \rangle = f_{\iota _{1},{\mathcal {I}}_{1}} \, f_{o,x_{\iota _{1}}} \det \big (B_{{\mathcal {I}}_{1}}\big ) \quad \text {and}\quad \det \langle H_{2} \rangle = f_{\iota _{2},{\mathcal {I}}_{2}} \, f_{\rho _{1},x_{\iota _{2}}} \det \big (B_{{\mathcal {I}}_{2}}\big ) \nonumber \\ \end{aligned}$$

where the blocks $B_{{\mathcal {I}}_{1}}$ and $B_{{\mathcal {I}}_{2}}$ are given by

$$\begin{aligned} B_{{\mathcal {I}}_{1}} = \left( \begin{array}{ccccc} f_{\iota _{2}, x_{\iota _{2}}} &{} \quad f_{\iota _{2}, x_{\rho _{1}}} &{} \quad f_{\iota _{2}, x_{\rho _{2}}} &{} \quad 0 &{} \quad 0 \\ f_{\rho _{1}, x_{\iota _{2}}} &{} \quad f_{\rho _{1}, x_{\rho _{1}}} &{} \quad f_{\rho _{1}, x_{\rho _{2}}} &{} \quad f_{\rho _{1}, x_{\rho _{3}}} &{} \quad f_{\rho _{1}, x_{\rho _{4}}} \\ 0 &{} \quad f_{\rho _{2}, x_{\rho _{1}}} &{} \quad f_{\rho _{2}, x_{\rho _{2}}} &{} \quad f_{\rho _{2}, x_{\rho _{3}}} &{} \quad f_{\rho _{2}, x_{\rho _{4}}} \\ 0 &{} \quad f_{\rho _{3}, x_{\rho _{1}}} &{} \quad f_{\rho _{3}, x_{\rho _{2}}} &{} \quad f_{\rho _{3}, x_{\rho _{3}}} &{} \quad 0 \\ 0 &{} \quad f_{\rho _{4}, x_{\rho _{1}}} &{} \quad f_{\rho _{4}, x_{\rho _{2}}} &{} \quad 0 &{} \quad f_{\rho _{4}, x_{\rho _{4}}}\end{array}\right) , \end{aligned}$$

and

$$\begin{aligned} B_{{\mathcal {I}}_{2}}= \left( \begin{array}{ccccc} f_{\iota _{1}, x_{\iota _{1}}} &{} \quad 0 &{} \quad f_{\iota _{1}, x_{\rho _{2}}} &{} \quad 0 &{} \quad 0 \\ 0 &{} \quad f_{\rho _{2}, x_{\rho _{1}}} &{} \quad f_{\rho _{2}, x_{\rho _{2}}} &{} \quad f_{\rho _{2}, x_{\rho _{3}}} &{} \quad f_{\rho _{2}, x_{\rho _{4}}} \\ 0 &{} \quad f_{\rho _{3}, x_{\rho _{1}}} &{} \quad f_{\rho _{3}, x_{\rho _{2}}} &{} \quad f_{\rho _{3}, x_{\rho _{3}}} &{} \quad 0 \\ 0 &{} \quad f_{\rho _{4}, x_{\rho _{1}}} &{} \quad f_{\rho _{4}, x_{\rho _{2}}} &{} \quad 0 &{} \quad f_{\rho _{4}, x_{\rho _{4}}} \\ f_{o, x_{\iota _{1}}} &{} \quad 0 &{} \quad f_{o, x_{\rho _{2}}} &{} \quad f_{o, x_{\rho _{3}}} &{} \quad 0 \end{array}\right) . \end{aligned}$$

Hence, the vector determinant is given by

3.3 $$\begin{aligned} {\widehat{h}} = \bigg ( f_{\iota _{1},{\mathcal {I}}_{1}} \, f_{o,x_{\iota _{1}}} \det \big (B_{{\mathcal {I}}_{1}}\big ), f_{\iota _{2},{\mathcal {I}}_{2}} \, f_{\rho _{1},x_{\iota _{2}}} \det \big (B_{{\mathcal {I}}_{2}}\big ) \bigg ). \end{aligned}$$

Note that by the structure of ${\widehat{h}}$ in (3.3), it is clear that the coordinates of ${\widehat{h}}$ have no common factor. Hence, according to Definition 2.9, the system does not support pleiotropic homeostasis. As by Proposition 2.8, the system must support in general some type of infinitesimal homeostasis, we conclude that it may present coincidental homeostasis. By our algorithm described in Sect. 2.8, this conclusion can also be derived directly from the analysis of the network corresponding to the dynamical system (3.1) (see Fig. 5).

We shall now list all the possible types of coincidental homeostasis that may happen. To simplify notation, we list the factor that appear in the coordinates of ${\widehat{h}}$ that may be equal to 0, and the corresponding classification of homeostasis.

1. $f_{o, x_{\iota _{1}}}$ (${\mathcal {I}}_{1}$-structural) and $f_{\rho _{1}, x_{\iota _{2}}}$ (${\mathcal {I}}_{2}$-structural);
2. $f_{o, x_{\iota _{1}}}$ (${\mathcal {I}}_{1}$-structural) and $\det \left( B_{{\mathcal {I}}_{2}} \right)$ (${\mathcal {I}}_{2}$-structural);
3. $\det \left( B_{{\mathcal {I}}_{1}} \right)$ (${\mathcal {I}}_{1}$-appendage) and $f_{\rho _{1}, x_{\iota _{2}}}$ (${\mathcal {I}}_{2}$-structural);
4. $\det \left( B_{{\mathcal {I}}_{1}} \right)$ (${\mathcal {I}}_{1}$-appendage) and $\det \left( B_{{\mathcal {I}}_{2}} \right)$ (${\mathcal {I}}_{2}$-structural).

The theoretical results above give the list of all possible homeostasis types of the general admissible system (3.1). As often, in a model-independent approach, we can not say much about what happens in a *specific model equation* of the form (3.1), such as the original model in Granjon et al. (2017). However, it may happen that some of the homeostasis types above do not occur in a *specific model equation*. For instance, it is easy to check if case (1) above can occur in a *specific model equation*: it is enough to compute $f_{o,x_1}$ and $f_{\rho _1,x_{\iota _2}}$ and verify that they never vanish. When this is the case, we can conclude that this homeostasis type cannot occur in that *specific model equation*. On the other hand, if they both can vanish, then it may be possible to find homeostasis points by numerical computation.

**Fig. 6:**
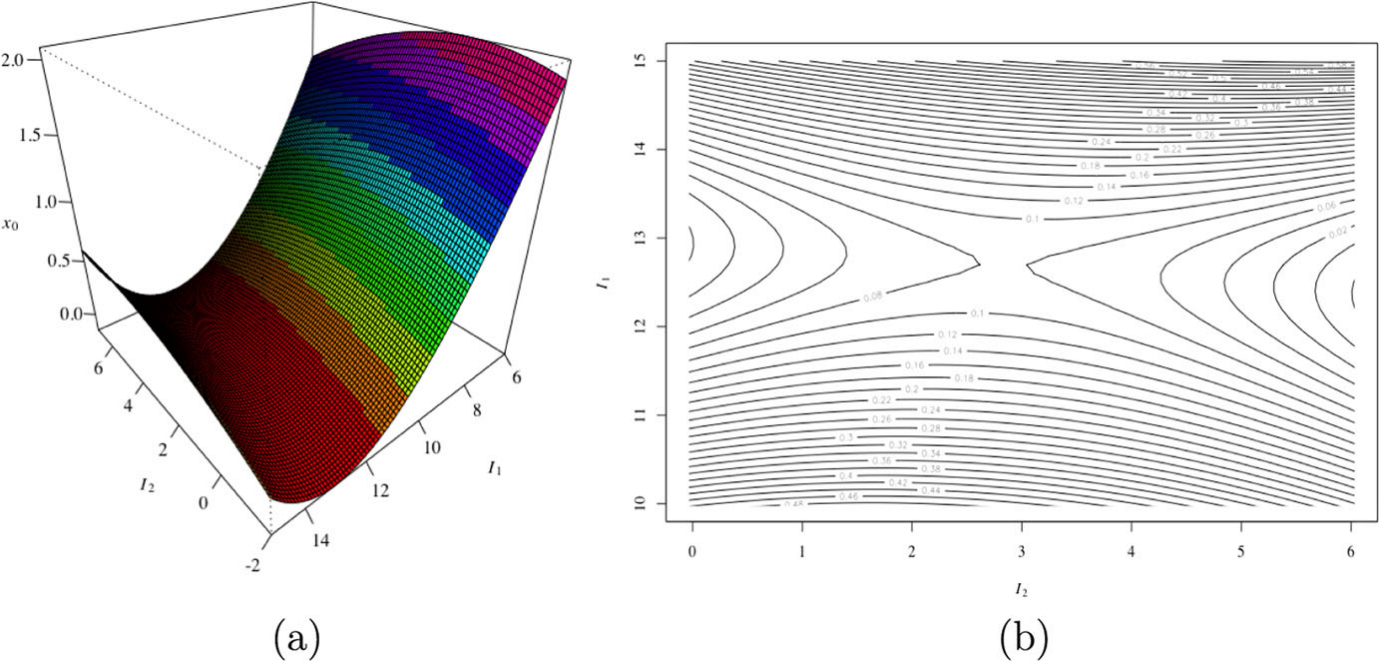
Numerical computation of the input–output function $x_o({\mathcal {I}}_1,{\mathcal {I}}_2)$ of a generic admissible vector field (3.1) truncated up to quadratic order. Infinitesimal homeostasis occurs at $({\mathcal {I}}_1^0, {\mathcal {I}}_2^0) \approx (2.9,12.7)$, with $x_o(2.9,12.7) \approx 0.09$. Panel **a** shows the 3D plot of the graph of $x_o({\mathcal {I}}_1,{\mathcal {I}}_2)$. Here, the scale of the *z*-axis ($x_o$) is different from the the scale of the other two axes. Panel **b** shows the contour plot (level curves) of $x_o({\mathcal {I}}_1,{\mathcal {I}}_2)$. Near the homeostasis point the function $x_o({\mathcal {I}}_1,{\mathcal {I}}_2)$ is topologically equivalent to *hyperbolic saddle*. The input–output function was numerically computed using xppaut (Ermentrout 2002) and plotted using r (R Core Team 2023)

In general, there is no obstruction for a ‘generic’ admissible system to display all possible homeostasis types. Moreover, it is not difficult to numerically find a point of infinitesimal homeostasis in a ‘generic’ admissible system. In Fig. 6 we present the result of a numeric computation of the input–output function $x_o({\mathcal {I}}_1,{\mathcal {I}}_2)$ of a generic admissible vector field (3.1) truncated up to quadratic order. The numeric computation allows us to find that infinitesimal homeostasis occurs at $({\mathcal {I}}_1^0, {\mathcal {I}}_2^0) \approx (2.9,12.7)$. The *plateau* is located at $x_o(2.9,12.7) \approx 0.09$. Near the singularity the function $x_o({\mathcal {I}}_1,{\mathcal {I}}_2)$ is topologically equivalent to a *hyperbolic saddle*—a *Morse singularity* in ${\mathbb {R}}^2$ with normal form $h({\mathcal {I}}_1, {\mathcal {I}}_2) = {\mathcal {I}}_1^2 -{\mathcal {I}}_2^2$ (see Golubitsky and Stewart (2018) for more details). Recall that a Morse singularity has codimension 0, thus it is *structurally stable*, namely any small perturbation of $x_o({\mathcal {I}}_1,{\mathcal {I}}_2)$ (induced by a small perturbation of the admissible vector field) is topologically equivalent to the unperturbed function. The *flatness* of the input–output function $x_o({\mathcal {I}}_1,{\mathcal {I}}_2)$ near the homeostasis point is reflected in the graph Fig. 6a, which shows that for $({\mathcal {I}}_1,{\mathcal {I}}_2) \in [6,15]\times [-2,7]$, the value of $x_o$ stays in [0, 2].

## Classification of homeostasis types

In this section we present the proofs of the main results of the paper.

### Reduction to the core network

In this section, unless explicitly stated, we assume that ${\mathcal {G}}$ is a multiple inputs network with input nodes $\iota _{1}, \ldots , \iota _{n}$, and inputs ${\mathcal {I}}_{1}, \ldots , {\mathcal {I}}_{N}$.

The definition of core subnetwork implies that the admissible system of Eqs. (2.17) can be written as

4.1 $$\begin{aligned} \begin{aligned}&f_{\iota _{1}}(x_{\iota _{1}}, \ldots , x_{\iota _{n}}, x_{\rho }, x_{d}, x_{o}, {\mathcal {I}}_{1},\ldots , {\mathcal {I}}_{N}) = 0 \\&\qquad \qquad \qquad \vdots \\&f_{\iota _{n}}(x_{\iota _{1}}, \ldots , x_{\iota _{n}}, x_{\rho }, x_{d}, x_{o}, {\mathcal {I}}_{1}, \ldots , {\mathcal {I}}_{N}) = 0 \\&f_{\rho }(x_{\iota _{1}}, \ldots , x_{\iota _{n}}, x_{\rho }, x_{d}, x_{o}) = 0 \\&f_{u}(x_{\iota _{1}}, \ldots , x_{\iota _{n}}, x_{\rho }, x_{u}, x_{d}, x_{o}) = 0 \\&f_{d}(x_{d}) = 0 \\&f_{o}(x_{\iota _{1}}, \ldots , x_{\iota _{n}}, x_{\rho }, x_{d}, x_{o}) = 0 \end{aligned} \end{aligned}$$

Now we can freeze the variables $x_{d}$ at an appropriate value and obtain an admissible system for ${\mathcal {G}}_{c}$ from system (4.1).

#### Lemma 4.1

Suppose that the point $X^* = (x_{\iota _{1}}^{*}, \ldots , x_{\iota _{n}}^{*}, x_{\rho }^{*}, x_{u}^{*}, x_{d}^{*}, x_{o}^{*})$ is a linearly stable equilibrium of (4.1). Then the ${\mathcal {G}}_c$-admissible system obtained from (4.1) by freezing $x_{d}$ at $x_{d}^{*}$, given by

4.2 $$\begin{aligned} \begin{aligned}&{\dot{x}}_{\iota _{1}} = f_{\iota _{1}}(x_{\iota _{1}}, \ldots , x_{\iota _{n}}, x_{\rho }, x_{d}^{*}, x_{o}, {\mathcal {I}}_{1},\ldots , {\mathcal {I}}_{N})\\&\qquad \qquad \qquad \vdots \\&{\dot{x}}_{\iota _{n}} = f_{\iota _{n}}(x_{\iota _{1}}, \ldots , x_{\iota _{n}}, x_{\rho }, x_{d}^{*}, x_{o}, {\mathcal {I}}_{1}, \ldots , {\mathcal {I}}_{N})\\&{\dot{x}}_{\rho } = f_{\rho }(x_{\iota _{1}}, \ldots , x_{\iota _{n}}, x_{\rho }, x_{d}^{*}, x_{o})\\&{\dot{x}}_{o} = f_{o}(x_{\iota _{1}}, \ldots , x_{\iota _{n}}, x_{\rho }, x_{d}^{*}, x_{o}) \end{aligned} \end{aligned}$$

has a linearly stable equilibrium $X_c^* = (x_{\iota _{1}}^{*}, \ldots , x_{\iota _{n}}^{*}, x_{\rho }^{*}, x_{o}^{*})$.

#### Proof

Clearly, $X_c^*$ is an equilibrium of (4.2). As shown in Madeira and Antoneli (2022, Lem 3.1), it is linearly stable. Indeed, the Jacobian matrix *J* of (4.1) evaluated at $X^*$ is

4.3 $$\begin{aligned} J = \begin{pmatrix} f_{\iota _{1}, x_{\iota _{1}}} &{} \quad \cdots &{} \quad f_{\iota _{1}, x_{\iota _{n}}} &{} \quad f_{\iota _{1}, x_{\rho }} &{} \quad f_{\iota _{1}, x_{d}} &{} \quad 0 &{} \quad f_{\iota _{1}, x_{o}} \\ \vdots &{} \quad \ddots &{} \quad \vdots &{} \quad \vdots &{} \quad \vdots &{} \quad \vdots &{} \quad \vdots \\ f_{\iota _{n}, x_{\iota _{1}}} &{} \quad \cdots &{} \quad f_{\iota _{n}, x_{\iota _{n}}} &{} \quad f_{\iota _{n}, x_{\rho }} &{} \quad f_{\iota _{n}, x_{d}} &{} \quad 0 &{} \quad f_{\iota _{n}, x_{o}} \\ f_{\rho , x_{\iota _{1}}} &{} \quad \cdots &{} \quad f_{\rho , x_{\iota _{n}}} &{} \quad f_{\rho , x_{\rho }} &{} \quad f_{\rho , x_{d}} &{} \quad 0 &{} \quad f_{\rho , x_{o}} \\ 0 &{} \quad \cdots &{} \quad 0 &{} \quad 0 &{} \quad f_{d, x_{d}} &{} \quad 0 &{} \quad 0 \\ f_{u, x_{\iota _{1}}} &{} \quad \cdots &{} \quad f_{u, x_{\iota _{n}}} &{} \quad f_{u, x_{\rho }} &{} \quad f_{u, x_{d}} &{} \quad f_{u, x_{u}} &{} \quad f_{u, x_{o}} \\ f_{o, x_{\iota _{1}}} &{} \quad \cdots &{} \quad f_{o, x_{\iota _{N}}} &{} \quad f_{o, x_{\rho }} &{} \quad f_{o, x_{d}} &{} \quad 0 &{} \quad f_{o, x_{o}} \end{pmatrix} \end{aligned}$$

Therefore, the eigenvalues of *J* are the same eigenvalues of $f_{d, x_{d}}$, $f_{u, x_{u}}$ and of the matrix $J_{c}$, where

4.4 $$\begin{aligned} J_{c} = \begin{pmatrix} f_{\iota _{1}, x_{\iota _{1}}} &{} \quad \cdots &{} \quad f_{\iota _{1}, x_{\iota _{n}}} &{} \quad f_{\iota _{1}, x_{\rho }} &{} \quad f_{\iota _{1}, x_{o}} \\ \vdots &{} \quad \ddots &{} \quad \vdots &{} \quad \vdots &{} \quad \vdots \\ f_{\iota _{n}, x_{\iota _{1}}} &{} \quad \cdots &{} \quad f_{\iota _{n}, x_{\iota _{n}}} &{} \quad f_{\iota _{n}, x_{\rho }} &{} \quad f_{\iota _{n}, x_{o}} \\ f_{\rho , x_{\iota _{1}}} &{} \quad \cdots &{} \quad f_{\rho , x_{\iota _{n}}} &{} \quad f_{\rho , x_{\rho }} &{} \quad f_{\rho , x_{o}} \\ f_{o, x_{\iota _{1}}} &{} \quad \cdots &{} \quad f_{o, x_{\iota _{n}}} &{} \quad f_{o, x_{\rho }} &{} \quad f_{o, x_{o}} \end{pmatrix} \end{aligned}$$

Since $J_{c}$ is the Jacobian matrix of (4.2) calculated at $X_c^*$, it follows that if $X^*$ is a linearly stable equilibrium then so it is $X_c^*$. $\square$

#### Theorem 4.2

Let $x_{o}({\mathcal {I}})$ be the input–output function of the admissible system 2.17 for the network ${\mathcal {G}}$ and let $x^{c}_{o}({\mathcal {I}})$ be the input–output function of the admissible system (4.2) for the corresponding core network ${\mathcal {G}}_c$. Then $x^{c}_{o}$ exhibits infinitesimal homeostasis at ${\mathcal {I}}^*$ if and only if $x_{o}$ exhibits infinitesimal homeostasis at ${\mathcal {I}}^*$.

#### Proof

For each weighted homeostasis matrix $\langle H_{M} \rangle$, we have:

4.5 $$\begin{aligned} \langle H_{M} \rangle = \begin{pmatrix} f_{\iota _{1}, x_{\iota _{1}}} &{} \quad \cdots &{} \quad f_{\iota _{1}, x_{\iota _{n}}} &{} \quad f_{\iota _{1}, x_{\rho }} &{} \quad f_{\iota _{1}, x_{d}} &{} \quad 0 &{} \quad - f_{\iota _{1}, {\mathcal {I}}_{M}} \\ \vdots &{} \quad \ddots &{} \quad \vdots &{} \quad \vdots &{} \quad \vdots &{} \quad \vdots &{} \quad \vdots \\ f_{\iota _{N}, x_{\iota _{1}}} &{} \quad \cdots &{} \quad f_{\iota _{N}, x_{\iota _{n}}} &{} \quad f_{\iota _{N}, x_{\rho }} &{} \quad f_{\iota _{N}, x_{d}} &{} \quad 0 &{} \quad - f_{\iota _{N}, {\mathcal {I}}_{M}} \\ f_{\rho , x_{\iota _{1}}} &{} \quad \cdots &{} \quad f_{\rho , x_{\iota _{n}}} &{} \quad f_{\rho , x_{\rho }} &{} \quad f_{\rho , x_{d}} &{} \quad 0 &{} \quad 0 \\ 0 &{} \quad \cdots &{} \quad 0 &{} \quad 0 &{} \quad f_{d, x_{d}} &{} \quad 0 &{} \quad 0 \\ f_{u, x_{\iota _{1}}} &{} \quad \cdots &{} \quad f_{u, x_{\iota _{n}}} &{} \quad f_{u, x_{\rho }} &{} \quad f_{u, x_{d}} &{} \quad f_{u, x_{u}} &{} \quad 0 \\ f_{o, x_{\iota _{1}}} &{} \quad \cdots &{} \quad f_{o, x_{\iota _{n}}} &{} \quad f_{o, x_{\rho }} &{} \quad f_{o, x_{d}} &{} \quad 0 &{} \quad 0 \end{pmatrix} \end{aligned}$$

Hence, for each $1 \le M \le N$, we have:

4.6 $$\begin{aligned} \det \langle H_{M} \rangle = \det (f_{d, x_{d}}) \det (f_{u, x_{u}}) \det \langle H^{c}_{M} \rangle \end{aligned}$$

where

4.7 $$\begin{aligned} \langle H^{c}_{M} \rangle = \begin{pmatrix} f_{\iota _{1}, x_{\iota _{1}}} &{} \quad \cdots &{} \quad f_{\iota _{1}, x_{\iota _{n}}} &{} \quad f_{\iota _{1}, x_{\rho }} &{} \quad - f_{\iota _{1}, {\mathcal {I}}_{M}} \\ \vdots &{} \quad \ddots &{} \quad \vdots &{} \quad \vdots &{} \quad \vdots \\ f_{\iota _{n}, x_{\iota _{1}}} &{} \quad \cdots &{} \quad f_{\iota _{n}, x_{\iota _{n}}} &{} \quad f_{\iota _{n}, x_{\rho }} &{} \quad - f_{\iota _{n}, {\mathcal {I}}_{M}} \\ f_{\rho , x_{\iota _{1}}} &{} \quad \cdots &{} \quad f_{\rho , x_{\iota _{n}}} &{} \quad f_{\rho , x_{\rho }} &{} \quad 0 \\ f_{o, x_{\iota _{1}}} &{} \quad \cdots &{} \quad f_{o, x_{\iota _{n}}} &{} \quad f_{o, x_{\rho }} &{} 0 \end{pmatrix} \end{aligned}$$

From Lemma 4.1, we have

4.8 $$\begin{aligned} \det (J) = \det (f_{d, x_{d}}) \, \det (f_{u, x_{u}}) \, \det (J_{c}) \end{aligned}$$

Applying (4.6) and (4.8) to (2.13), we get:

4.9 $$\begin{aligned} \nabla x_{o} = \frac{1}{\det (J_{c})} \big (\det \langle H^{c}_{1} \rangle , \det \langle H^{c}_{2} \rangle , \ldots , \det \langle H^{c}_{N} \rangle \big ) = \nabla x^c_{o} \end{aligned}$$

Therefore, $x_{o}$ and $x^c_{o}$ have exactly the same critical points. $\square$

### Classification of pleiotropic homeostasis types

In this sub-section, unless explicitly stated, we assume that ${\mathcal {G}}$ is a core multiple inputs network with input nodes $\iota _{1}, \ldots , \iota _{n}$, and inputs ${\mathcal {I}}_{1}, \ldots , {\mathcal {I}}_{N}$, with $n,N \ge 2$.

We shall now study the subnetworks associated to pleiotropic homeostasis blocks. Bearing this in mind, we start by extending the classification of nodes from Madeira and Antoneli (2022).

#### Definition 4.1

Let ${\mathcal {G}}$ be a multiparameter core network.

- A directed path connecting nodes $\rho$ and $\tau$ is called a *simple path* if it visits each node on the path at most once.
- An $\iota _{m}o$-*simple path* is a simple path connecting the input node $\iota _{m}$ to the output node *o*.
- A node is $\iota _{m}$-*simple* if it lies on an $\iota _{m}o$-simple path.
- A node is $\iota _{m}$-*appendage* if it is downstream from $\iota _{m}$ and it is not an $\iota _{m}$-simple node.
- A node is ${\mathcal {I}}_{M}$-*absolutely simple* if it is an $\iota _{m}$-simple node, for every *m* such that $f_{\iota _{m}, {\mathcal {I}}_{M}} \not \equiv 0$.
- A node is ${\mathcal {I}}_{M}$-*absolutely appendage* if it is an $\iota _{m}$-appendage node, for every *m* such that $f_{\iota _{m}, {\mathcal {I}}_{M}} \not \equiv 0$.
- An $\iota _{m}$-*super-simple node* is an $\iota _{m}$-simple node that lies on every $\iota _{m}o$-simple path.
- An ${\mathcal {I}}_{M}$-*absolutely super-simple node* is a node that lies on every $\iota _{m}o$-simple path, for every *m* such that $f_{\iota _{m}, {\mathcal {I}}_{M}} \not \equiv 0$. $\Diamond$

It is immediate that the output node *o* is an ${\mathcal {I}}_{M}$-absolutely super-simple node, for all $M = 1, \ldots , N$.

#### Pleiotropic-appendage homeostasis

To study the structure of pleiotropic-appendage homeostasis, we shall first generalize the concepts of path equivalence and appendage subnetworks employed in Wang et al. (2021), Madeira and Antoneli (2022) to the current context.

##### Definition 4.2

Let ${\mathcal {K}}$ be a nonempty subnetwork of ${\mathcal {G}}$. We say that nodes $\rho _{i}, \rho _{j}$ of ${\mathcal {K}}$ are *path equivalent in* ${\mathcal {K}}$ (or ${\mathcal {K}}$-*path equivalent*) if there are paths in ${\mathcal {K}}$ from $\rho _{i}$ to $\rho _{j}$ and from $\rho _{j}$ to $\rho _{i}$. A ${\mathcal {K}}$*-path component* is a path equivalence class in ${\mathcal {K}}$. $\Diamond$

##### Definition 4.3

The ${\mathcal {G}}$*-complementary subnetwork* of an $\iota _{m}o$-simple path *S* is the subnetwork *CS* consisting of all nodes of ${\mathcal {G}}$ not on *S* and all arrows in ${\mathcal {G}}$ connecting those nodes. $\Diamond$

##### Definition 4.4

Let ${\mathcal {G}}$ be a multiparameter core network.

- For every $m = 1, \ldots , n$, we define the $\iota _{m}$-*appendage subnetwork* ${\mathcal {A}}_{{\mathcal {G}}_{m}}$ as the subnetwork of ${\mathcal {G}}$ composed by all $\iota _{m}$-appendage nodes and all arrows in ${\mathcal {G}}$ connecting $\iota _{m}$-appendage nodes.
- For every $M = 1, \ldots , N$, we define the ${\mathcal {I}}_{M}$-*appendage subnetwork* ${\mathcal {A}}_{{\mathcal {G}}_{{\mathcal {I}}_{M}}}$ as the subnetwork of ${\mathcal {G}}$ composed by all ${\mathcal {I}}_{M}$-absolutely appendage nodes and all arrows in ${\mathcal {G}}$ connecting ${\mathcal {I}}_{M}$-absolutely appendage nodes. That is,
  $$\begin{aligned} {\mathcal {A}}_{{\mathcal {G}}_{{\mathcal {I}}_{M}}} = \bigcap _{\begin{array}{c} 1 \le m \le n: \\ f_{\iota _{m}, {\mathcal {I}}_{M}} \not \equiv 0 \end{array}} {\mathcal {A}}_{{\mathcal {G}}_{m}}. \end{aligned}$$
- The *appendage subnetwork* ${\mathcal {A}}_{{\mathcal {G}}}$ is the subnetwork of ${\mathcal {G}}$ composed by nodes which are ${\mathcal {I}}_{M}$-absolutely appendage, for all $M = 1, \ldots , N$, and the arrows connecting such nodes. That is,
  $$\begin{aligned} {\mathcal {A}}_{{\mathcal {G}}} = \bigcap _{1 \le M \le N } {\mathcal {A}}_{{\mathcal {G}}_{{\mathcal {I}}_{M}}} = \bigcap _{1 \le m \le n} {\mathcal {A}}_{{\mathcal {G}}_{m}}. \end{aligned}$$

Now we can characterize the structure of pleiotropic-appendage homeostasis. Let *B* be a pleiotropic appendage block. By a similar argument employed in Madeira and Antoneli (2022), we conclude that *B* must be the jacobian matrix of the corresponding subnetwork ${\mathcal {K}}_{B}$.

##### Theorem 4.3

Let ${\mathcal {K}}_{B}$ be a subnetwork of ${\mathcal {G}}$ associated with a pleiotropic-appendage block *B*. Then the following statements are valid:

(i) Each node in ${\mathcal {K}}_{B}$ is an $I_{M}$-absolutely appendage node, for all $M = 1, \ldots , N$.
(ii) For every $\iota _{m}o$-simple path *S*, nodes in ${\mathcal {K}}_{B}$ are not *CS*-path equivalent to any node in $CS{\setminus } {\mathcal {K}}_{B}$, for all $m = 1, \ldots , n$;
(iii) ${\mathcal {K}}_{B}$ is a path component of ${\mathcal {A}}_{{\mathcal {G}}}$.

##### Proof

Statements (*a*) and (*b*) follow by applying (Madeira and Antoneli 2022, Thm 3.11) to each of the core subnetworks ${\mathcal {G}}_{{\mathcal {I}}_{M}}$. Statement (*c*) is proved along the same line as (Madeira and Antoneli 2022, Thm 3.11c). $\square$

Now we shall verify that the conditions listed in Theorem 4.3 are also sufficient to guarantee the existence of a pleiotropic-appendage homeostasis block.

##### Theorem 4.4

Suppose ${\mathcal {K}}$ is a subnetwork of ${\mathcal {G}}$ such that:

(i) ${\mathcal {K}}$ is an ${\mathcal {A}}_{{\mathcal {G}}}$-path component;
(ii) For every $\iota _{m}o$-simple path *S*, nodes in ${\mathcal {K}}$ are not *CS*-path equivalent to any node in $CS{\setminus } {\mathcal {K}}_{j}$, for all $m = 1, \ldots , n$.

Then $\det (J_{{\mathcal {K}}})$ is an irreducible factor of ${\widehat{h}}$.

##### Proof

Apply (Madeira and Antoneli 2022, Thm 3.13) to each of the specialized subnetworks ${\mathcal {G}}_{I_{m}}$. The validity of condition (*b*) of (Madeira and Antoneli 2022, Thm 3.13) for each specialized subnetwork ${\mathcal {G}}_{{\mathcal {I}}_{M}}$ follows directly of condition (*b*) of this theorem. It is then enough to prove that ${\mathcal {K}}$ is a path component of ${\mathcal {A}}_{{\mathcal {G}}_{{\mathcal {I}}_{M}}}$, for all $m = 1, \ldots , n$. As ${\mathcal {K}}_{j}$ is a path component of ${\mathcal {A}}_{{\mathcal {G}}} = \bigcap _{1 \le M \le N } {\mathcal {A}}_{{\mathcal {G}}_{{\mathcal {I}}_{M}}}$, then for each $M = 1, \ldots , N$, there is a ${\mathcal {A}}_{{\mathcal {G}}_{{\mathcal {I}}_{M}}}$-path component ${\mathcal {T}}_{M}$ such that ${\mathcal {K}} \subseteq {\mathcal {T}}_{M}$. By condition (*b*), it follows that ${\mathcal {K}} = {\mathcal {T}}_{M}$, for each $M = 1, \ldots , N$. $\square$

#### Pleiotropic-structural homeostasis

Now we shall study the pleiotropic-structural blocks.

Let ${\mathcal {V}}^{{\mathcal {G}}}$ be the set of nodes of ${\mathcal {G}}$, ${\mathcal {V}}^{{\mathcal {G}}}_{\iota }$ the set of nodes that are $\iota _{m}$-super simple, for all $m = 1, \ldots , n$ and ${\mathcal {V}}^{{\mathcal {G}}}_{{\mathcal {I}}}$ the set of nodes that are ${\mathcal {I}}_{M}$-absolutely super-simple, for all $M = 1, \ldots , N$. In Madeira and Antoneli (2022), we introduced the notion of absolutely super-simple nodes with respect to the input nodes. This suggests that we can define absolutely super-simple nodes with respect to the inputs. This leads to the question: Which subset of ${\mathcal {V}}^{{\mathcal {G}}}$ is more suitable to base the characterization of pleiotropic-structural subnetworks:${\mathcal {V}}^{{\mathcal {G}}}_{\iota }$ or ${\mathcal {V}}^{{\mathcal {G}}}_{I}$. The simple, yet paramount, observation that the answer to this question is that both sets are equal.

##### Lemma 4.5

Let ${\mathcal {G}}$ be a multiple inputs core network. Then ${\mathcal {V}}^{{\mathcal {G}}}_{\iota } = {\mathcal {V}}^{{\mathcal {G}}}_{{\mathcal {I}}}$.

##### Proof

It is enough to verify that ${\mathcal {V}}^{{\mathcal {G}}} {\setminus } {\mathcal {V}}^{{\mathcal {G}}}_{\iota } = {\mathcal {V}}^{{\mathcal {G}}} {\setminus } {\mathcal {V}}^{{\mathcal {G}}}_{{\mathcal {I}}}$. First, suppose there is a node $\rho \in {\mathcal {V}}^{{\mathcal {G}}} {\setminus } {\mathcal {V}}^{{\mathcal {G}}}_{\iota }$. Then, there is at least one input node $\iota _{m}$ such that $\rho$ is not an $\iota _{m}$-super-simple node. As ${\mathcal {G}}$ is a core network, there is an input ${\mathcal {I}}_{M}$ such that $f_{I_{M}, \iota _{m}} \not \equiv 0$, which implies that $\rho$ is not ${\mathcal {I}}_{M}$-absolutely super-simple and hence $\rho \in {\mathcal {V}}^{{\mathcal {G}}} {\setminus } {\mathcal {V}}^{{\mathcal {G}}}_{{\mathcal {I}}}$. On the other hand, if $\rho \not \in {\mathcal {V}}^{{\mathcal {G}}}_{{\mathcal {I}}}$, then there exists *M* such that $\rho$ is not ${\mathcal {I}}_{M}$-absolutely super-simple $\Rightarrow \rho$ is not $\iota _{m}$-super-simple, for some *m* such that $f_{{\mathcal {I}}_{M}, \iota _{m}} \not \equiv 0 \Rightarrow \rho \not \in {\mathcal {V}}^{{\mathcal {G}}}_{\iota }$. $\square$

The importance of Lemma 4.5 is that it allows us to study the set ${\mathcal {V}}^{{\mathcal {G}}}_{\iota } = {\mathcal {V}}^{{\mathcal {G}}}_{I}$ through either the characterization with respect to the input nodes or to the inputs, whichever is more convenient. In particular, we can easily extend many of the results obtained in Madeira and Antoneli (2022).

A slightly modification of the argument of Lemma 4.5 shows that the set of nodes that are $\iota _{m}$-simple, for all $m = 1, \ldots , n$, and the set of nodes ${\mathcal {I}}_{M}$-absolutely simple, for all $M = 1, \ldots , N$, are also equal. These observations justify the generalization of the concept of absolutely simple and absolutely super-simple nodes.

##### Definition 4.5

Let ${\mathcal {G}}$ be a multiparamete core network.

- A node $\rho$ is called *absolutely super-simple* if and only if it is an $\iota _{m}$-super simple node, for all $m = 1, \ldots , n$. Equivalently, $\rho$ is called *absolutely super-simple* if and only if it is an $I_{M}$-absolutely super-simple node, for all $M = 1, \ldots , N$.
- A node $\rho$ is called *absolutely simple* if and only if it is an $\iota _{m}-$simple node, for all $m = 1, \ldots , n$. Equivalently, $\rho$ is called *absolutely simple* if and only if it is an $I_{M}$-absolutely simple node, for all $M = 1, \ldots , N$. $\Diamond$

Following Madeira and Antoneli (2022), we now define a total order relation in the set of absolutely super-simple nodes.

##### Definition 4.6

Let ${\mathcal {G}}$ be a multiparamete core network. Define a relation on ${\mathcal {V}}^{{\mathcal {G}}}_{\iota } = {\mathcal {V}}^{{\mathcal {G}}}_{{\mathcal {I}}}$ as follows: for any pair of nodes $\sigma , \tau \in {\mathcal {V}}^{{\mathcal {G}}}_{\iota } = {\mathcal {V}}^{{\mathcal {G}}}_{{\mathcal {I}}}$, $\sigma \ne \rho$, we write $\sigma > \rho$ when $\rho$ is downstream from $\sigma$ by all $\iota _{m}o$-simple paths, for any $m = 1, \ldots , n$. $\Diamond$

##### Lemma 4.6

The relation on ${\mathcal {V}}^{{\mathcal {G}}}_{\iota } = {\mathcal {V}}^{{\mathcal {G}}}_{{\mathcal {I}}}$ given in Definition 4.6 is a total order.

##### Proof

This result is analogous to (Madeira and Antoneli 2022, Lem 3.15) $\square$

Consider now the ordered elements of ${\mathcal {V}}^{{\mathcal {G}}}_{\iota }$: $\rho _{1}> \rho _{2}> \cdots> \rho _{p} > o$. Similarly to Wang et al. (2021); Madeira and Antoneli (2022), we say that two elements $\rho _{k} > \rho _{k+1}$ of ${\mathcal {V}}^{{\mathcal {G}}}_{\iota }$ are *adjacent* when $\rho _{k+1}$ is the first element of ${\mathcal {V}}^{{\mathcal {G}}}_{\iota }$ which appears after $\rho _{k}$ in that ordering. We can now use this concept to introduce the elements that are crucial to characterise pleiotropic-structural homeostasis blocks.

##### Definition 4.7

Let $\rho _{k} > \rho _{k+1}$ be adjacent elements of ${\mathcal {V}}^{{\mathcal {G}}}_{\iota }$. An $\iota _{m}$-absolutely simple node $\rho$ is *between* $\rho _{k}$ and $\rho _{k+1}$ if there exists an $\iota _{w}o$-simple path that includes $\rho _{k}$ to $\rho$ to $\rho _{k+1}$ in that order, for some $w = 1,\ldots ,n$. $\Diamond$

The idea is to construct the structural subnetworks employing the concepts above, as it was done in Wang et al. (2021); Madeira and Antoneli (2022).

The *absolutely super-simple subnetwork*, denoted ${\mathcal {L}}(\rho _{k}, \rho _{k+1})$, is the subnetwork whose nodes are absolutely simple nodes between $\rho _{k}$ and $\rho _{k+1}$ and whose arrows are arrows of ${\mathcal {G}}$ connecting nodes in ${\mathcal {L}}(\rho _{k}, \rho _{k+1})$. As we can characterise the absolutely super-simple and absolutely simple nodes (and consequently the absolutely super-simple subnetworks) with respect to each input node, we can construct the basic unit of pleiotropic-structural homeostasis in the same way the basic unit of structural homeostasis was constructed in Madeira and Antoneli (2022).

##### Definition 4.8

Let $\rho _{k}$ and $\rho _{k+1}$ be adjacent absolutely super-simple nodes in ${\mathcal {G}}$. The *absolutely super-simple structural subnetwork* ${\mathcal {L}}'(\rho _{k}, \rho _{k+1})$ is the input–output subnetwork consisting of nodes in ${\mathcal {L}}(\rho _{k}, \rho _{k+1}) \cup {\mathcal {B}}$, where ${\mathcal {B}}$ consists of all absolutely appendage nodes that are $CS_{m}$-path equivalent to nodes in ${\mathcal {L}}(\rho _{k}, \rho _{k+1})$ for some $\iota _{m}o$-simple path $S_{m}$, for some $m \in \{1,\ldots ,n\}$. That is, ${\mathcal {B}}$ consists of all ${\mathcal {A}}_{{\mathcal {G}}}$-path components ${\mathcal {B}}_{i}$ that are $CS_{m}$-path equivalent to nodes in ${\mathcal {L}}(\rho _{k}, \rho _{k+1})$ for some $S_{m}$, for some $m \in \{1,\ldots ,n\}$. Arrows of ${\mathcal {L}}'(\rho _{k}, \rho _{k+1})$ are arrows of ${\mathcal {G}}$ that connect nodes in ${\mathcal {L}}'(\rho _{k}, \rho _{k+1})$. Note that $\rho _{k}$ is the input node and that $\rho _{k+1}$ is the output node of ${\mathcal {L}}'(\rho _{k}, \rho _{k+1})$. $\Diamond$

We shall employ the characterisation of super-simple structural subnetworks with respect to each of the input nodes. This was the strategy employed in Madeira and Antoneli (2022), and hence we will be able to apply directly the results contained in Madeira and Antoneli (2022, Sect. 3.4.2) to the case of networks with multiple inputs.

First, for $\rho _{k}$ and $\rho _{k+1}$ adjacent $\iota _{m}$-super-simple nodes in the core subnetwork ${\mathcal {G}}_{m}$, define as in Madeira and Antoneli (2022) the $\iota _{m}$*-super-simple structural subnetwork* ${\mathcal {L}}'_{m}(\rho _{k}, \rho _{k+1})$ as the input–output subnetwork consisting of nodes in ${\mathcal {L}}_{m}(\rho _{k}, \rho _{k+1}) \cup {\mathcal {B}}_{m}$, where ${\mathcal {B}}_{m}$ consists of all $\iota _{m}$-appendage nodes that are $C_{m}S_{m}$-path equivalent to nodes in ${\mathcal {L}}_{m}(\rho _{k}, \rho _{k+1})$ for some $\iota _{m}o$-simple path $S_{m}$. As usual, arrows of ${\mathcal {L}}'_{m}(\rho _{k}, \rho _{k+1})$ are arrows of ${\mathcal {G}}_{m}$ that connect nodes in ${\mathcal {L}}'_{m}(\rho _{k}, \rho _{k+1})$.

We notice that (Madeira and Antoneli 2022, Lemma 3.21) is still valid in the current context. Hence, we obtain the following.

##### Lemma 4.7

Let $\rho _{k}>\rho _{k+1}$ be two adjacent absolutely super-simple nodes. Then ${\mathcal {L}}'_{m}(\rho _{k}, \rho _{k+1}) = {\mathcal {L}}'(\rho _{k}, \rho _{k+1})$, for every $m=1,\ldots ,n$.

##### Theorem 4.8

Let ${\mathcal {K}}_{B}$ be a subnetwork of ${\mathcal {G}}$ associated with a pleiotropic-structural block *B*. Then ${\mathcal {G}}$ has adjacent absolutely super-simple nodes $\rho _{k}$ and $\rho _{k + 1}$ such that ${\mathcal {K}}_{B} = {\mathcal {L}}'(\rho _{k}, \rho _{k + 1})$.

##### Proof

If *B* is an irreducible pleiotropic-structural block, then it is a structural block associated to each specialized subnetwork ${\mathcal {G}}_{{\mathcal {I}}_{M}}$. Fix an input ${\mathcal {I}}_{M}$ and consider the corresponding specialized subnetwork ${\mathcal {G}}_{{\mathcal {I}}_{M}}$. By Madeira and Antoneli (2022, Thm 3.22), (Golubitsky and Wang 2020, Thm 6.11) and Lemma 4.7, this implies that there exist $\iota _{m}$-absolutely super-simple nodes $\rho _{k_{M}}$ and $\rho _{k_{M} + 1}$ such that ${\mathcal {K}}_{B} = {\mathcal {L}}_{m}'(\rho _{k_{M}}, \rho _{k_{M} + 1})$ for all *m* such that $\iota _{m}$ is an input node of the specialized subnetwork ${\mathcal {G}}_{{\mathcal {I}}_{M}}$. Now, as the input and output nodes of all these networks must be the same, we conclude that there exist absolutely super-simple nodes $\rho _{k}, \rho _{k+1}$ such that for all $m = 1, \ldots , n$, ${\mathcal {K}}_{B} = {\mathcal {L}}_{m}'(\rho _{k}, \rho _{k + 1})$. By Lemma 4.7, this means that ${\mathcal {K}}_{B} = {\mathcal {L}}'(\rho _{k}, \rho _{k + 1})$. $\square$

The argument in the proof of Theorem 4.8 suggests that, as in the case of input–output networks with only one input (Madeira and Antoneli 2022; Golubitsky and Wang 2020), a multiple inputs input–output network supports pleiotropic-structural homeostasis whenever there are more than one absolutely super-simple node.

##### Theorem 4.9

If ${\mathcal {G}}$ has absolutely super-simple nodes other than the output node, then each absolutely super-simple structural subnetwork corresponds to a pleiotropic-structural homeostasis subnetwork.

##### Proof

Consider the adjacent absolutely super-simple nodes $\rho _{k}, \rho _{k+1}$ in ${\mathcal {G}}$. By Lemma 4.5, for every $m=1,\ldots ,n$, we have ${\mathcal {L}}'_{m}(\rho _{k}, \rho _{k+1}) = {\mathcal {L}}'(\rho _{k}, \rho _{k+1})$. As proved in Madeira and Antoneli (2022, Cor 3.23), this means that the homeostasis matrix of ${\mathcal {L}}'(\rho _{k}, \rho _{k+1})$ is an irreducible structural homeostasis subnetwork of each ${\mathcal {I}}_{M}$-specialized subnetwork ${\mathcal {G}}_{{\mathcal {I}}_{M}}$. Therefore the homeostasis matrix of ${\mathcal {L}}'(\rho _{k}, \rho _{k+1})$ is an irreducible pleiotropic-structural homeostasis subnetwork. $\square$

## Conclusion and outlook

In this paper, we present a framework for the analysis and classification of homeostasis types multiple input single output networks. We accomplish this by generalizing and extending the results of Wang et al. (2021) and Madeira and Antoneli (2022) for the classification of homeostasis types in single-input networks single-output networks. Wang et al. (2021) treat the case where the single input parameter affects a single input node and Madeira and Antoneli (2022) consider the case where the single input parameter may affect multiple input nodes.

In the terminology of Golubitsky and Stewart (2022) our theory is an example of a *model independent* approach. This means that the classification results obtained here provide a complete list of possible behaviors, with respect to homeostasis, that is *independent* of the model equations—the list depends only on the topology of the network. Which of those behaviors will be observed in a particular realization of the dynamics (e.g. a model equation) *depends* on the specific form of the dynamics.

We illustrate the application of the theory in several examples. In Sect. 2.9, Example 2.15, we analyze the simplest class of multiple inputs networks: the two inputs, three node networks, where each input node is affected by exactly one input parameter (see Fig. 3). In Example 2.16 we have a two inputs, three node network violating this condition—namely, with more than one input parameter affecting the same input node (see Fig. 4). Finally, in Sect. 3 we consider a biologically realistic model for the co-regulation of calcium and phosphate (Granjon et al. 2017) (see Fig. 5).

In three of the eight networks in Fig. 3—cases (b), (e) and (f)—and the network in Fig. 4, we found the ‘simultaneous occurrence’ of infinitesimal homeostasis and steady-state bifurcation, for certain coincidental homeostasis types (see Remark 2.17). Strictly speaking, this kind of behavior is forbidden by definition, because at a bifurcation point the input–output function becomes ill-defined. However, it is possible, in certain situations, to extend the definition of input–output function to allow for the presence of singular points (see Duncan et al. 2018; Duncan and Golubitsky 2019). These extensions of the notion of homeostasis open up the door for a rich variety of phenomena. For instance, in Mulukutla et al. (2014) the authors investigate glycolysis metabolism and discover a switch mechanism based on a bistability phenomena occurring simultaneously with homeostasis.

The systematic blending of homeostasis and steady-state bifurcations seems to be a promising research avenue. In this regard, the infinitesimal homeostasis approach has some benefit due to its singularity theoretic flavor and the fact that there exists a mature theory of bifurcations based on singularity theory (Golubitsky and Schaeffer 1985; Golubitsky et al. 1988). In fact, Duncan and Golubitsky (2019) is, in part, an attempt to explain the observations of Mulukutla et al. (2014) using singularity theory to uncover the ‘interaction’ between homeostasis and steady-state bifurcations.

In our examples it seems that the interaction between homeostasis and steady-state bifurcations is ‘caused’ by the overlapping of the subnetworks associated to certain coincidental blocks in distinct components of the vector determinant. This is distinct from the phenomena discovered in Duncan et al. (2023), where it is shown that an interaction between homeostasis and steady-state bifurcations may occur already in single input node, single input parameter networks.

Regarding the classification of homeostasis types, we were able to completely characterize the pleiotropic homeostasis types and have provided necessary and sufficient conditions for its occurrence (Sect. 4). The main result essentially says that pleiotropic homeostasis types are exactly the homeostasis types that occur in single input parameter, single input node networks.

As for the coincidental homeostasis type, the situation is much more complex. On one hand, were able to obtain some sufficient conditions for its occurrence (see Proposition 2.9). On the other hand, we have given examples where only pleotropic types can occur (Proposition  2.6) and examples where only coincidental types can occur (Example 2.16, cases (a), (b), (c), (d), (f) and the network for calcium and phosphate homeostasis). Furthermore, by Proposition  2.8 any core network must have at least one homeostasis type. Which implies that if there is no coincidental homeostasis type the all homeostasis types must be pleiotropic. All these considerations suggest that a necessary and sufficient condition for occurrence of coincidental types seems rather elusive (see Remark 2.14) and is an important open problem at the moment.
